# Lysine benzoylation of fatty acid β-oxidation core enzyme FoxA regulates the aflatoxin biosynthesis by benzoyltransferase EsaA in the pathogenic fungus *Aspergillus flavus*

**DOI:** 10.1128/mbio.03672-25

**Published:** 2026-04-20

**Authors:** Xuan Chen, Shuqi Huang, Zhiwei Jiang, Yuqi Zhang, Shihua Wang

**Affiliations:** 1 State Key Laboratory of Agricultural and Forestry Biosecurity, Key Laboratory of Pathogenic Fungi and Mycotoxins of Fujian Province, and School of Life Sciences, Fujian Agriculture and Forestry University 528652, Fuzhou, China; 2 Key Laboratory of Infection and Immunity, Health Commission of Jiangxi Province, School of Basic Medicine, Nanchang Medical College 649550, Nanchang, China; University of Toronto, Toronto, ON, Canada

**Keywords:** benzoylation, FoxA, EsaA, aflatoxin, *Aspergillus flavus*

## Abstract

Lysine benzoylation (Kbz) is a newly identified post-translational modification, which participates in the regulation of a variety of cellular processes. However, the function of Kbz in pathogenic fungi remains unclear. Here, we first identified that FoxA is a benzoylated protein with two benzoylated sites at lysines 425 and 433. Mutations of Kbz sites in FoxA significantly reduced long-chain fatty acid (LCFA) utilization activity and exhibited a phenotype similar to that of *foxA* gene deletion mutant, including decreased conidiation and aflatoxin production, reduced seed colonization, and increased sclerotia formation. Metabolomic analyses indicated that the deletion of FoxA or interference with its benzoylation could disrupt peroxisomal β-oxidation, resulting in the accumulation of LCFAs. This disruption may inhibit conidiation and aflatoxin production by modulating the synthesis of 15d-PGJ(2)-G. Furthermore, we found that EsaA has benzoyltransferase activity *in vitro* and *in vivo*, and its expression influences the Kbz of FoxA and LCFA utilization activity. Notably, mutants at the acetylated sites exhibited phenotypes similar to those of the benzoylated site mutants, suggesting that acetylation also plays a significant role in FoxA protein. Our study uncovers a previously unknown mechanism by which benzoylation and acetylation regulate FoxA activity to affect the development, secondary metabolism, and pathogenicity of *A. flavus*.

**IMPORTANCE:**

As a predominantly plant-pathogenic fungus, *Aspergillus flavus* not only causes severe crop diseases and economic losses but also poses a global food safety threat due to its production of aflatoxins. Furthermore, under certain conditions, it can act as an opportunistic pathogen, endangering the health of both humans and animals. Numerous studies have shown that protein post-translational modifications, such as acetylation, succinylation, and benzylation, were involved in aflatoxin production; however, the exact mechanism was still unclear. This study reveals, for the first time, the molecular mechanism by which benzoylation regulates the functional execution of multifunctional β-oxidation hydratase/dehydrogenase FoxA proteins and further influences the development and aflatoxin synthesis in *Aspergillus flavus*. This discovery not only provides new insights for the prevention and control of aflatoxin contamination but also provides theoretical support for the study of secondary metabolism regulation mechanisms of other fungi.

## INTRODUCTION

Post-translational modification (PTM) refers to the covalent modification of proteins, which serves as a key mechanism for diversifying their functions (1, 2). A classic example is traditional acetylation, which broadly governs critical cellular processes, such as transcription, cell division, and neural function (1). In recent years, an increasing number of newly PTMs are identified, including benzoylation (2), lactylation (3), isonicotinylation (4), and methacrylation (5). Among these, lysine benzoylation (Kbz) is the only acylation that has a benzene ring group, which enables it to effectively neutralize the positive charge of lysine and induce a greater size change and stronger hydrophobicity in comparison to lysine acetylation (Kac) (2). Consistent with acetylation, benzoylation occurs on both histone benzoylated sites and non-histone benzoylated proteins (6, 7). Numerous studies indicate that histone Kbz is required for activating gene transcription and some metabolism (2, 6), while benzoylated non-histone proteins have been shown to participate in diverse biological processes, including glycolysis/gluconeogenesis and ribosome biogenesis (7). Currently, Kbz is found in yeast, RAW cells, and HepG2 cells (2, 6, 7), and our previous data also found that Kbz exists in the fungus *A. flavus* (8).

*Aspergillus flavus* is a conditionally pathogenic fungus that is widely distributed in natural environments (9). *A. flavus* can behave as an opportunistic pathogen able to determine Aspergillosis and fungal keratitis infections (10, 11). On the other hand, as a plant-pathogenic fungus, *A. flavus* contamination not only causes great economic losses but also seriously harms human health (12). Meanwhile, *A. flavus* produces a series of mycotoxins, such as aflatoxins, cyclopiazonic acid, and aflatrem, which can result in serious threats to human health and cause enormous economic losses in farming (13). Of these mycotoxins, the most abundant and toxic secondary metabolite is aflatoxin B_1_ (AFB_1_), which is one of the most toxic natural contaminants (14). Since aflatoxin can be bioaccumulated in the human body through the food chain, aflatoxin contamination of crops becomes a global food safety concern (15). Given the significant detrimental effects of *A. flavus* and aflatoxin, it is imperative to investigate the regulatory mechanisms of pathogenicity and elucidate the regulatory network involved in aflatoxin biosynthesis in *A. flavus*.

Acetyl coenzyme A (acyl-CoA) was a precursor for aflatoxin biosynthesis, which can be produced via the glycolytic pathway, pyruvate dehydrogenase bypass pathway, and fatty acid β-oxidation (16, 17). The peroxisomal β-oxidation consists of four main steps, in which long-chain fatty acids (LCFAs) undergo dehydrogenation (acyl-CoA oxidase Fox1p-catalyzed), hydration and dehydrogenation (enzoyl-CoA hydratase and 3-hydroxyacyl-CoA dehydrogenase Fox2p-catalyzed), and sulfur cleavage (3-ketoacyl-CoA thiolase Fox3p-catalyzed) to produce acyl-CoA (17, 18). Numerous studies demonstrate that peroxisomal β-oxidation plays an important role in mycotoxin biosynthesis; for example, oleic acid, as a LCFA, could induce aflatoxin production in *A. flavus* (19). Deficiency of *foxA* (Fox2p homologs) leads to a significant decrease in sterigmatocystin production of *Aspergillus nidulans* on maize kernels (20), but overexpression of *foxA* could result in increased aflatoxin production of *A. flavus* (21). Recent studies have demonstrated that acylation plays a role in modulating the structure and function of modified proteins (22, 23), and our previous findings identify potential benzoylation modification in FoxA protein (8). However, the role of FoxA benzoylation in the regulation of aflatoxin production remains unknown.

The purpose of this study was to explore the role of benzoylation in FoxA protein. We performed Western blot (WB) and phenotypic assays to reveal that benzoylated protein FoxA was important in fungal development, aflatoxin synthesis, and pathogenicity in *A. flavus*. We also found that benzoylation defects of FoxA lead to reduced functional activity and further affect development, aflatoxin biosynthesis, and pathogenicity. Furthermore, we proved that EsaA was a benzoyltransferase for FoxA. Meanwhile, acetylation plays an important role in FoxA functional execution. Therefore, we concluded that benzoylation and acetylation could regulate FoxA function, thus modulating the development and aflatoxin biosynthesis of *A. flavus*. This is the first functional study to reveal the roles of benzoylation and acetylation in FoxA, providing new insights into the prevention and control of fungal disease.

## RESULTS

### Identification of a novel Kbz protein FoxA in *A. flavus*

**Fig 1:**
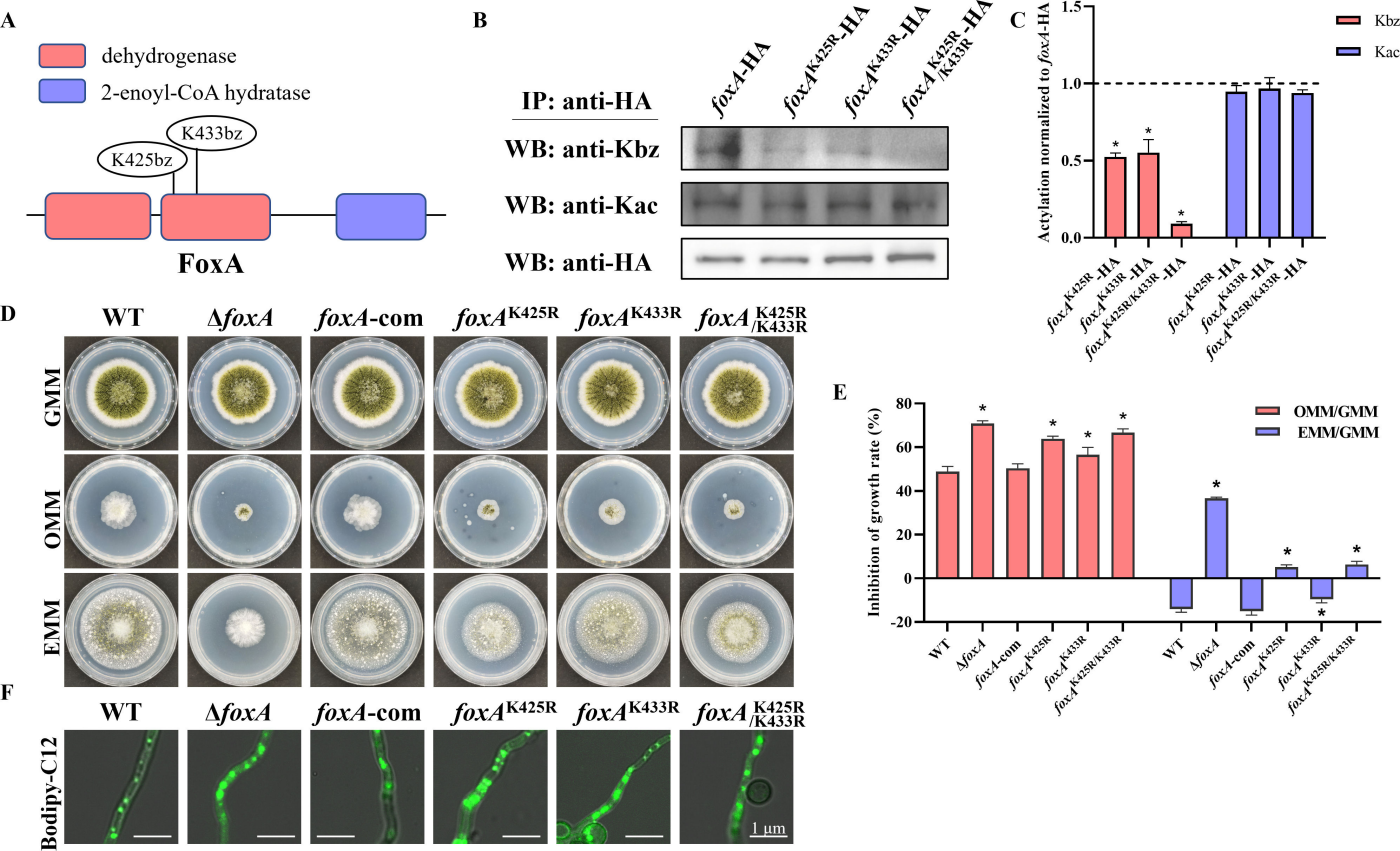
Benzoylated protein FoxA is required for fatty acid β-oxidation and aflatoxin biosynthesis. (**A**) Schematic drawing of the functional domains and modified site of FoxA in *A. flavus*. (**B**) Densitometric quantification of the Kbz levels of FoxA in WT and *foxA* mutants as determined via immunoprecipitation and immunoblotting. (**C**) Relative Kbz and Kac levels in *foxA*-HA mutants. (**D**) Colony morphology of WT and *foxA* mutants grown on GMM, OMM, or EMM media at 37°C for 5 days. (**E**) The inhibition rate of WT and *foxA* mutants (*n* = 3). Asterisks represent statistically significant differences (*P* < 0.05). (**F**) Microscopy of WT and *foxA* mutants stained with BODIPY-C12 for 2 h. Scale bar = 1 µm.

Multifunctional β-oxidation hydratase/dehydrogenase (FoxA) was a β-oxidation core enzyme, which was crucial in the regulation of fatty acid and energy metabolism (24). The FoxA of *A. flavus* encodes 900 amino acids, which contains two conserved dehydrogenase domains and one 2-enoyl-CoA hydratase domain. Our previous benzoylomic results identified two benzoylated sites on FoxA protein, located at K425 and K433 (Fig. 1A; Fig. S1A and B). Sequence alignment analysis demonstrated that the benzoylated lysine residues K425 and K433 were evolutionary conserved among different fungi (Fig. S1C). To validate benzoylomic sequencing data of FoxA, a FoxA-HA fusion mutant was generated. The WB results showed that FoxA-HA strain exhibited Kbz signal (Fig. 1B), indicating that FoxA was benzoylated protein in *A. flavus*.

### FoxA deletion impairs LCFA utilization, aflatoxin production, and fungal development

To investigate the functions of FoxA in *A. flavus*, gene deletion (Δ*foxA*) and complementation (*foxA*-com) mutants were constructed and verified (Fig. S2A through C). While the Δ*foxA* mutation significantly restricted growth on erucic acid (C22:1) and oleic acid (C18:1), as assessed by vegetative growth on solid medium, reintroduction of the *foxA* gene could restore wild-type (WT) levels of *A. flavus* growth on solid medium with different carbon sources (Fig. 1D and E). Using Bodipy-C12 staining to localize peroxisomes (25), we observed that the peroxisomes were enlarged in the Δ*foxA* mutant compared to the WT strain (Fig. 1F). These results demonstrated that FoxA was involved in β-oxidation in *A. flavus*.

Previous studies showed that fatty acids β-oxidation could promote aflatoxin formation (20), and the FoxA mediates the degradation of long-chain fatty acids into short-chain fatty acids. The deletion of *foxA* leads to a reduction in the formation of short-chain fatty acids, which may subsequently inhibit the production of acetyl-CoA and malonyl-CoA in *A. flavus*. Given that acetyl-CoA and malonyl-CoA serve as precursors for aflatoxin synthesis, we speculated that the deletion of *foxA* may affect aflatoxin production. To verify the hypothesis, we detected the aflatoxin production in WT and FoxA mutants, and thin-layer chromatography (TLC) analysis revealed a marked decrease in aflatoxin production when the *foxA* gene was absent (Fig. S2D and E).

Meanwhile, we also incubated spores of WT and FoxA mutants in YGT agar medium and CM agar medium. The phenotypic results showed that the Δ*foxA* mutant exhibited decreased conidiation and increased sclerotium production (Fig. S2F through I). To confirm the obtained phenotype, we examined the expression level of conidiation regulatory genes (*abaA* and *wetA*) and sclerotium regulatory gene (*nsdC*). As expected, the result showed that *abaA* and *wetA* were significantly decreased in the Δ*foxA* strain, but *nsdC* was significantly increased in the Δ*foxA* strain (Fig. S2J and K). These results indicated that FoxA was crucial for β-oxidation, aflatoxin production, and development in *A. flavus*.

### Mutation of benzoylated sites K425 and K433 inhibits LCFA utilization activity in *A. flavus*

FoxA has three important domains, including two dehydrogenase domains and a 2-enoyl-CoA hydratase domain, and candidate Kbz sites were located on the second dehydrogenase domain. To validate the benzoylated sites of FoxA protein, point mutants *foxA*^K425R^-HA, *foxA*^K433R^-HA, and *foxA*^K425R/433R^-HA were generated (Fig. S3A), in which arginine (R) substitution was used to mimic unacylated forms of lysine (K). The HA antibody was used for immunoprecipitation (IP), and the IP product was analyzed by immunoblotting with pan-Kbz and pan-Kac antibody. Compared with the *foxA*-HA strain, the Kbz signal of *foxA*^K425R^-HA and *foxA*^K433R^-HA strains was reduced, while the Kbz signal in the *foxA*^K425R/433R^-HA strain was nearly undetectable (Fig. 1B and C). Concurrently, no significant differences were observed in the acetylation levels among the different FoxA mutants (Fig. 1B and C). These results confirmed that K425 and K433 of FoxA were indeed benzoylated sites in *A. flavus*.

FoxA was a crucial enzyme in the fatty acid metabolism, which was responsible for the degradation of long-chain fatty acids (26). To evaluate the effect of benzoylation on the FoxA, we compared the LCFA utilization activity and peroxisome size among the WT, Δ*foxA*, *foxA*^K425R^, *foxA*^K433R^, and *foxA*^K425R/433R^ strains. Compared to the WT, both the benzoylated site mutants (*foxA*^K425R^, *foxA*^K433R^, and *foxA*^K425R/433R^) and the Δ*foxA* mutant exhibited higher inhibition rates (Fig. 1D and E; Fig. S3B), along with significantly enlarged peroxisomes (Fig. 1F; Fig. S3B). This indicates that defects in FoxA benzoylation lead to impaired long-chain fatty acid utilization. Furthermore, localization results showed that FoxA consistently co-localized with peroxisomes in both the *foxA*-HA and *foxA*^K425R/433R^-HA strains (Fig. S4). These findings demonstrate that K425 and K433 are critical benzoylation sites regulating FoxA function, and that mutations at benzoylated sites can directly affect FoxA activity.

### Mutation of benzoylated sites in FoxA caused decreased aflatoxin production and pathogenicity

**Fig 2:**
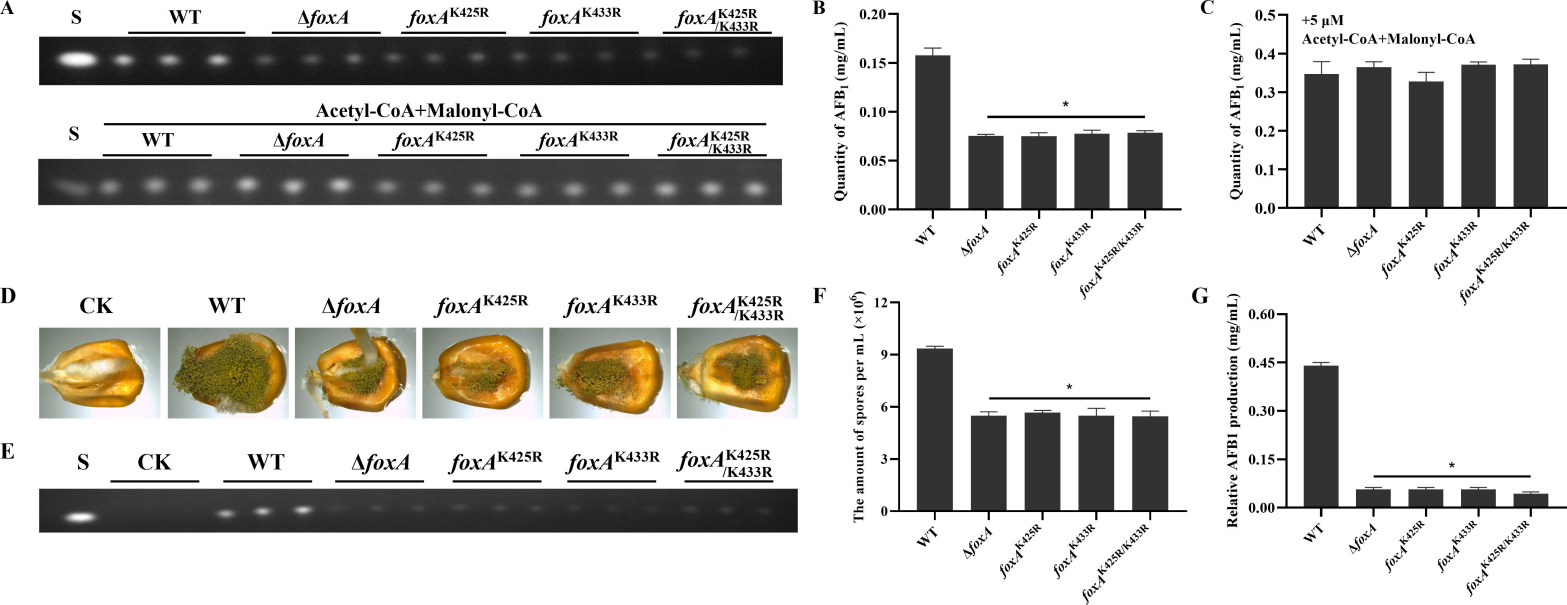
Kbz of FoxA was essential for fatty acid β-oxidation and aflatoxin production. (**A**) TLC assay of AFB_1_ production by WT and *foxA* mutants cultured in media or in GMM liquid media supplemented with 2.5 μM acetyl-CoA and 2.5 μM malonyl-CoA. S indicates the AFB1 standard. (**B**) Quantification of AFB_1_ in TLC results by optical density (*n* = 3). (**C**) Quantification of AFB_1_ in TLC results from media supplemented with acetyl-CoA and malonyl-CoA (*n* = 3). (**D**) Phenotypic observation of WT and *foxA* mutants in maize kernels. (**E**) TLC assay was used to detect the AFB_1_ production extracted from the infected maize kernels (*n* = 3). (**F**) Quantification of conidia from the infected maize kernels (*n* = 3). (**G**) Quantification of AFB_1_ in TLC results by optical density (*n* = 3). Asterisks represent statistically significant differences (*P* < 0.05).

Aflatoxin was a critical virulence factor in *A. flavus*, and its biosynthetic precursor acetyl-CoA could be provided by fatty acid generated from β-oxidation (27), which implied that Kbz may regulate the aflatoxin synthesis by affecting the activity of FoxA in *A. flavus*. To validate this hypothesis, we quantified the AFB_1_ production in WT, Δ*foxA*, *foxA*^K425R^, *foxA*^K433R^, and *foxA*^K425R/433R^ strains. Compared with WT, all *foxA* mutants produced fewer AFB_1_ (Fig. 2A and B). Subsequently, we supplemented the medium with acetyl-CoA and malonyl-CoA. The results showed that CoA supplementation could directly rescue the toxin reduction caused by *foxA* gene knockout or acyl-site mutations (Fig. 2A through C; Fig. S5), indicating that FoxA deficiency or acyl-site mutation reduces aflatoxin production by inhibiting short-chain fatty acid formation. Furthermore, these findings revealed that the benzoylated sites of FoxA were involved in regulating aflatoxin biosynthesis in *A. flavus*.

In the natural environment, the plant pathogenic fungus *A. flavus* mainly colonizes and contaminates crops through produce spores and aflatoxins (28). To determine the effects of FoxA and its benzoylation on pathogenicity, live maize seeds were inoculated with spores from the WT and *foxA* mutants, and fungal burden, conidial number, and aflatoxin production were examined. Controls (CK) were inoculated with sterile water. We found that *foxA* deletion and benzoylated site mutants significantly reduced the AFB_1_ production, conidia production, and fungal DNA burden (Fig. 2D thorugh G; Fig. S6). These results indicated that *foxA* and its Kbz site contribute to seed colonization in *A. flavus*.

### Mutation of benzoylated site in FoxA leads to a decrease in conidiation but an increase in sclerotium formation

Our results showed that FoxA positively regulates conidiation but negatively regulates sclerotium formation. To further investigate the potential roles of Kbz sites of FoxA in conidiation and sclerotia formation of *A. flavus*, WT and *foxA* mutants (Δ*foxA*, *foxA*^K425R^, *foxA*^K433R^, and *foxA*^K425R/433R^) were incubated in GMM agar media and CM agar media at 37°C. The phenotypic results showed that benzoylated site mutants exhibited similar phenotype to *foxA* deletion mutant, including decreased colony diameter, reduced conidial production, and increased sclerotial amounts (Fig. S7). These results indicated that benzoylated sites K425 and K433 could modulate fungal development by regulating the FoxA activity in *A. flavus*.

### Analysis of non-targeted metabolomics

FoxA is a metabolic enzyme whose altered protein activity should affect intracellular metabolism in *A. flavus*. To explore FoxA and its benzoylation in fungal metabolism, the differential metabolomics analysis between WT, Δ*foxA*, and *foxA*^K425R/K433R^ strains was carried out. The quality control results indicated that the response intensities and retention times of the peaks basically overlapped, suggesting that the instrument was stabilized with small instrument error (Fig. S8A and B). The PCA diagram indicated good repeatability and clear separation (Fig. S8C). In total, we identified 1,803 known metabolites, of which the majority were organic acids and derivatives (29.1%), lipids and lipid-like molecules (21%), and organoheterocyclic compounds (11%) (Fig. S8D). All the raw data of the metabolome were uploaded to the China National Gene Bank database (CNP0006675).

Based on univariate analysis, differential metabolites were analyzed in positive and negative ion modes, and metabolites with FC >1.5 or FC < 0.67, and *P* value< 0.05 were set as differential metabolites. Compared to WT, 1,969 upregulated metabolites and 339 downregulated metabolites were detected in the Δ*foxA* strain in positive ion mode, while 2,986 upregulated metabolites and 187 downregulated metabolites were detected in negative ion mode (Fig. S9A and B). Meanwhile, in positive ion mode, 1,843 upregulated metabolites and 124 downregulated metabolites were detected in the *foxA*^K425R/433R^ strain compared to WT, while 1,166 upregulated metabolites and 125 downregulated metabolites were detected in negative ion mode (Fig. S9C and D). Further analysis of the identified metabolites showed that the Δ*foxA* strain identified 117 and 78 known differential metabolites in positive and negative ion mode compared with WT, while the *foxA*^425R/K433R^ strain identified 85 and 25 known differential metabolites in positive and negative ion mode, respectively (Fig. S9E and F). The above results suggested that FoxA and its benzoylation were closely involved in various metabolic processes in *A. flavus*.

To clarify the role of FoxA and its benzoylation in the metabolic regulatory network of *A. flavus*, we analyzed the KEGG pathway annotation of differential metabolites. The differential metabolites of *foxA* gene deletion were mainly enriched in amino acid metabolism, lipid metabolism, carbohydrate metabolism, and other secondary metabolite synthesis (Fig. S10A). However, differential metabolites of FoxA benzoylation deficiency were mainly enriched in signal transduction, amino acid metabolism, and carbohydrate metabolism pathways (Fig. S10B). In addition, amino acid metabolism, arginine metabolism, and ABC protein transport pathways were significantly co-enriched in FoxA mutants (Fig. S10C and D), suggesting that FoxA and its benzoylation participate in metabolism regulation of *A. flavus*.

### Metabolomic data revealed FoxA, and its benzoylation was required for aflatoxin production and fatty acid β-oxidation

**Fig 3:**
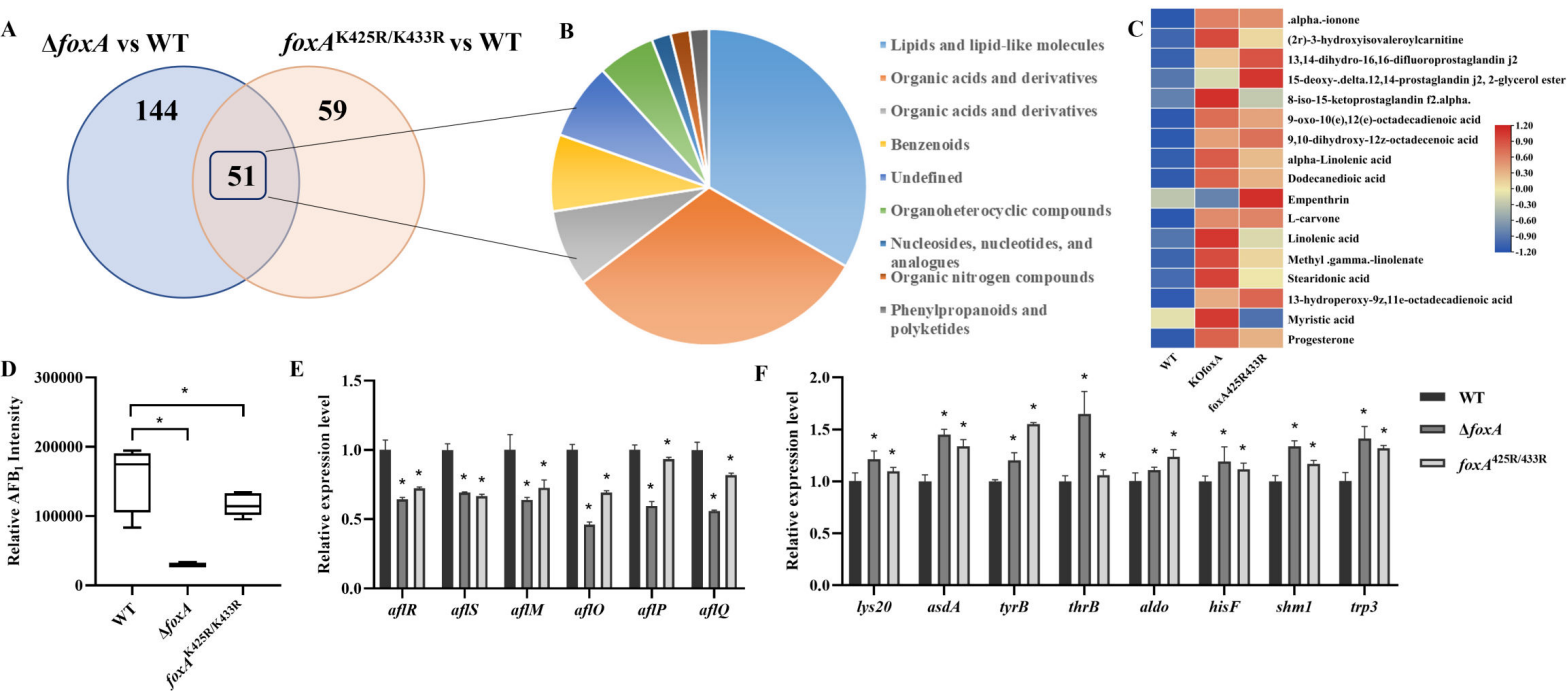
The role of FoxA and its benzoylation in fatty acid β-oxidation and aflatoxin biosynthesis. (**A**) Venn diagram of differential metabolites in Δ*foxA* and *foxA*^425R/433R^ strain. (**B**) Co-metabolite classification statistics. (**C**) The clustering heatmap of differential metabolites lipid and lipid-like molecules. (**D**) Relative content of aflatoxin AFB1. (**E**) Relative transcript levels of aflatoxin biosynthesis associated genes in different strains (*n* = 3). (**F**) Relative expression level of *lys20*, *asdA*, *tyrB*, *thrB*, *aldo*, *hisF*, *shm1,* and *trp3* genes (*n* = 3). Asterisks represent statistically significant differences (*P* < 0.05).

The comparative metabolomic data revealed 195 differential metabolites between the Δ*foxA* and WT strains, and 110 between the *foxA*^K425R/K433R^ and WT strains. Notably, 51 metabolites were common to both sets (Fig. 3A), underscoring the specific contribution of benzoylation to FoxA function. Among these, 17 differential metabolites were lipids and lipid-like molecules (Fig. 3B and C), such as alpha-linolenic acid, dodecanedioic acid, 9,10-dihydroxy-12z-octadecenoic acid, and stearidonic acid. Of the co-differential lipids and lipid-like molecules, 15 molecules exhibited higher content in Δ*foxA* and *foxA*^K425R/K433R^ compared to WT (Fig. 3C). These results indicated that deletion of *foxA* and its benzoylation would hinder fatty acid β-oxidation and impede the efficient degradation of LCFAs, ultimately resulting in the intracellular accumulation of LCFAs.

Subsequently, the metabolomic results showed that AFB_1_ production was decreased in Δ*foxA* and *foxA*^K425R/433R^ strains (Fig. 3D), which was similar to previous TLC results (Fig. 2E). Consistently, the qPCR data showed that the transcript levels of the AFB_1_ cluster genes were all decreased in the Δ*foxA* and *foxA*^K425R/433R^ compared with those in the WT (Fig. 3E). These results demonstrated that FoxA and its benzoylation indeed participate in the regulation of aflatoxin biosynthesis.

In order to explore the regulatory mechanisms of metabolic pathways, the transcriptional expression levels of enzymes involved in amino acid synthesis, such as homocitrate *lys20*, aspartate 4-decarboxylase *asdA*, aromatic-amino-acid transaminase *tyrB*, homoserine kinase *thrB*, fructose-bisphosphate aldolase *aldo*, imidazole glycerol-phosphate synthase subunit *hisF*, glycine hydroxymethyltransferase *shm1*, and anthranilate synthase/indole-3-glycerol phosphate synthase *trp3*, were further examined by qRT-PCR. The above genes were significantly upregulated in both Δ*foxA* and *foxA*^K425R/433R^ mutants compared to the WT strain (Fig. 3F), which is consistent with the result of the metabolomic assay. Collectively, these results demonstrate that the deletion of the *foxA* gene disrupts not only fatty acid β-oxidation but also has profound and far-reaching consequences on the broader metabolic network of *A. flavus*.

### Increased metabolite 15d-PGJ(2)-G induced defect growth, conidiation, and aflatoxin production

**Fig 4:**
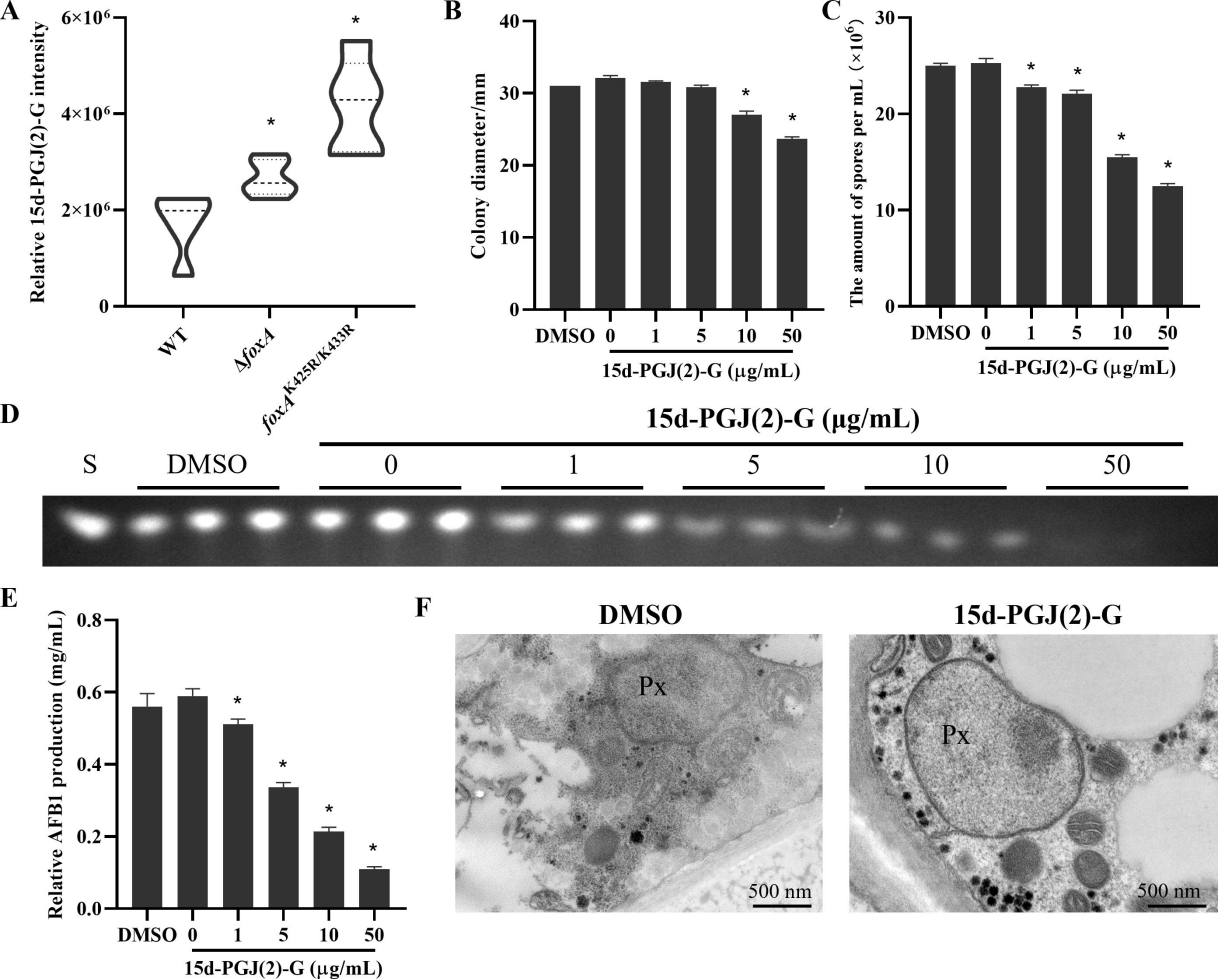
Effect of 15d-PGJ(2)-G in growth, conidiation, and aflatoxin production of *A. flavus*. (**A**) Relative content of 15d-PGJ(2)-G. (**B and C**) Colony morphology (**B**) and conidiation (**C**) of *A. flavus* with 15d-PGJ(2)-G treatment at different concentrations. (**D**) TLC analysis AFB_1_ production of *A. flavus* with sodium benzoate treatment at different concentrations (*n* = 3). (**E**) Quantification of AFB_1_ in TLC results by optical density (*n* = 3). Asterisks represent statistically significant differences (*P* < 0.05). (**F**) Electron microscopy analysis was performed on WT grown on medium containing DMSO or 15d-PGJ(2)-G. Scale bar = 500 nm. Asterisks represent statistically significant differences (*P* < 0.05).

FoxA is a key enzyme in peroxisomal fatty acid metabolism, and its absence leads to abnormal expansion of the peroxisome (29). Among the significantly different metabolites, 15-deoxy-delta-12,14-prostaglandin j2, 2-glycerol ester [15d-PGJ(2)-G] was found to be significantly upregulated in Δ*foxA* and *foxA*^K425R/433R^ relative to the WT strain (Fig. 4A). In addition, previous studies proved that 15d-PGJ(2)-G was involved in peroxisome proliferation (30), suggesting that metabolite 15d-PGJ(2)-G may play an important role in the execution of FoxA biological functions. To verify our hypothesis, we inoculated the WT strain into YGT medium containing different concentrations of 15d-PGJ(2)-G (0–50 μg/mL). The phenotypic results showed a dose-dependent decrease of growth rate, conidiation, and aflatoxin production after 15d-PGJ(2)-G treatment (Fig. 4B through E). The phenotype induced by 15d-PGJ(2)-G was similar to *foxA* gene deletion strain, and previous studies have shown that defects in peroxisome β-oxidation exhibit a changed peroxisome size and abundance, so we hypothesized that 15d-PGJ(2)-G might also affect the peroxisome morphology. Electron microscopy revealed that peroxisomes in 15d-PGJ(2)-G treatment appeared larger in size than peroxisomes in untreated WT cells (Fig. 4F). These results reflected that FoxA and its benzoylation may regulate the development and aflatoxin production of *A. flavus* by mediating 15d-PGJ(2)-G production.

### EsaA has benzoyltransferase activity *in vivo* and *in vitro*

Previous research has demonstrated that EsaA has benzoyltransferase activity in *Saccharomyces cerevisiae* (7). To explore whether EsaA has similar benzoyltransferase activity in *A. flavus*, we constructed the *esaA* deletion and overexpression mutants. Considering that numerous studies proved that EsaA was essential for vegetative growth in eukaryotic cells, such as *S. cerevisiae* and *A. nidulans* (31, 32)*,* we cannot directly knockout the fully *esaA* gene in the genome of *A. flavus*. Our real-time qPCR data showed that the copies of the *esaA* gene were two-fold higher than that of the *AfsumO* gene, which served as a single copy control (33) (Fig. S11A); therefore, we generated a single-copy deletion mutant *esaA*^−/+^ and overexpression mutant OE::*esaA* for enzymatic assays (Fig. S11B through D).

**Fig 5:**
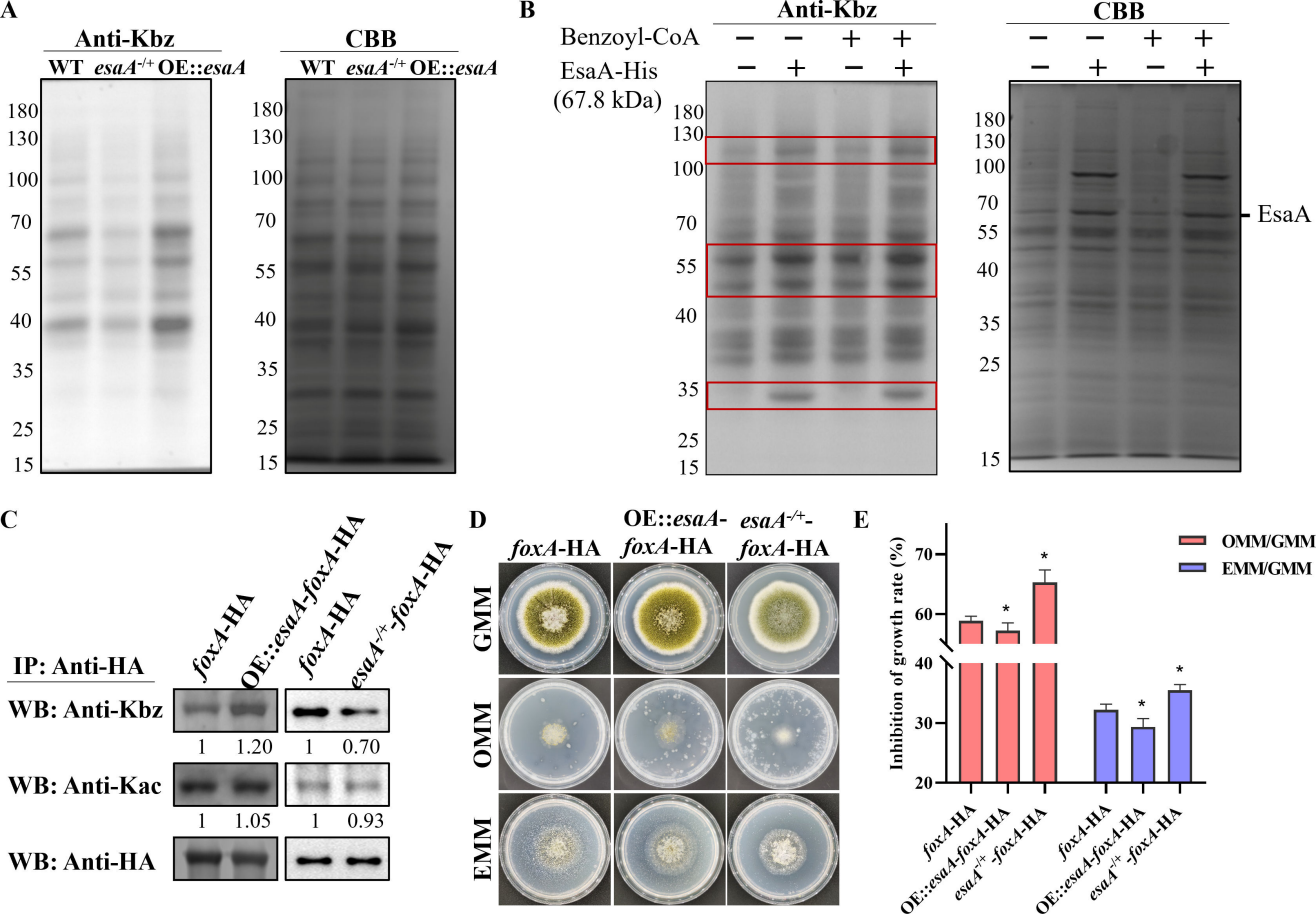
EsaA is involved in benzoylation of FoxA protein. (**A**) Western blot analysis of Kbz in WT, *esaA*^−/+^, and OE::*esaA* strains. (**B**) *In vitro* benzoyltransferase activity of EsaA by Western blotting analysis. (**C**) Densitometric quantification of the Kbz and Kac levels of FoxA protein in *foxA*-HA, OE::*esaA-foxA*-HA, and *esaA^−/+^-foxA*-HA strains as determined via immunoprecipitation and immunoblotting. (**D**) Colony morphology of *foxA*-HA, OE::*esaA-foxA*-HA, and *esaA^−/+^-foxA*-HA strains grown on GMM, OMM, or EMM media at 37°C for 5 days. (**E**) The inhibition rate of *foxA*-HA, OE::*esaA-foxA*-HA, and *esaA^−/+^-foxA*-HA strains (*n* = 3).

To study potential benzoyltransferase activity of EsaA in *A. flavus*, we performed WB assays to examine Kbz level of WT, *esaA*^−/+^, and OE::*esaA* strains *in vivo*. The results showed that Kbz level was significantly downregulated in the *esaA*^−/+^ mutant but upregulated in the OE::*esaA* mutant (Fig. 5A), indicating that *esaA* has benzoyltransferase activity in *A. flavus*. EsaA was the major member of the NuA4 complex, and the role of the NuA4 complex in Kbz is unclear, so we further constructed single deletion (Δ*eaf6* and Δ*yng2*) and inducible repression (*epl1*^xylP^) mutants of this complex (Fig. S12A). The Kbz level was decreased in *esaA*^−/+^ mutant and increased in OE::*esaA* mutants significantly, but no changes in other complex member mutants (Δ*eaf6,* Δ*yng2*, and *epl1*^xylP^) (Fig. S12B and C), suggesting that EsaA catalyzes benzoylation in a NuA4 complex-independent manner.

To examine whether EsaA has benzoyltransferase activity *in vitro*, we expressed AflEsaA and obtained the purified proteins (Fig. S13). We assessed the *in vitro* benzoyltransferase activity of EsaA by measuring the benzoylation levels in cell lysates after the addition of purified EsaA protein and/or benzoyl-CoA. The results showed that Kbz signal was increased in some protein bands with EsaA-His and benzoyl-CoA treatment, but it did not happen with only benzoyl-CoA treatment (Fig. 5B), which suggests that EsaA functions as a benzoyltransferase *in vitro*.

### EsaA was involved in benzoylation of FoxA

In yeast, it was demonstrated that both GcnE and EsaA exhibit benzoyltransferase activity (7). To test whether EsaA or GcnE could mediate the benzoylation of FoxA, we constructed OE::*esaA-foxA*-HA, *esaA^−/+^-foxA*-HA, and *gcnE*^xylP^-*foxA*-HA (Fig. S14), and then examined the Kbz and Kac signals of FoxA in different HA fusion mutants (*foxA*-HA, OE::*esaA-foxA*-HA, *esaA^−/+^-foxA*-HA, and *gcnE*^xylP^-*foxA*-HA). The results showed that both overexpression and repression of GcnE had no impact on the acetylation or benzoylation levels of FoxA protein (Fig. S15). As expected, overexpression of EsaA increased the benzoylation level of endogenous FoxA, and repression of EsaA decreased the benzoylation level of FoxA, but did not affect the acetylation level of FoxA (Fig. 5C). Hence, we speculated that EsaA could directly regulate the endogenous FoxA activity. To validate our speculation, we performed the LCFA utilization assay in *foxA*-HA, OE::*esaA-foxA*-HA, and *esaA^−/+^-foxA*-HA strains. The results showed that overexpression of *esaA* significantly rescued the growth defect observed in the FoxA mutant (Fig. 5D and E). In contrast, single-copy knockout of *esaA* exacerbated the growth inhibition on LCFA medium (Fig. 5D and E). These results further supported that EsaA was the benzoyltransferase of FoxA in *A. flavus*, and EsaA modulates fatty acid β-oxidation by regulating benzoylation of FoxA.

### Both CHO domain and MOZ domain are important in benzoyltransferase activity of EsaA

**Fig 6:**
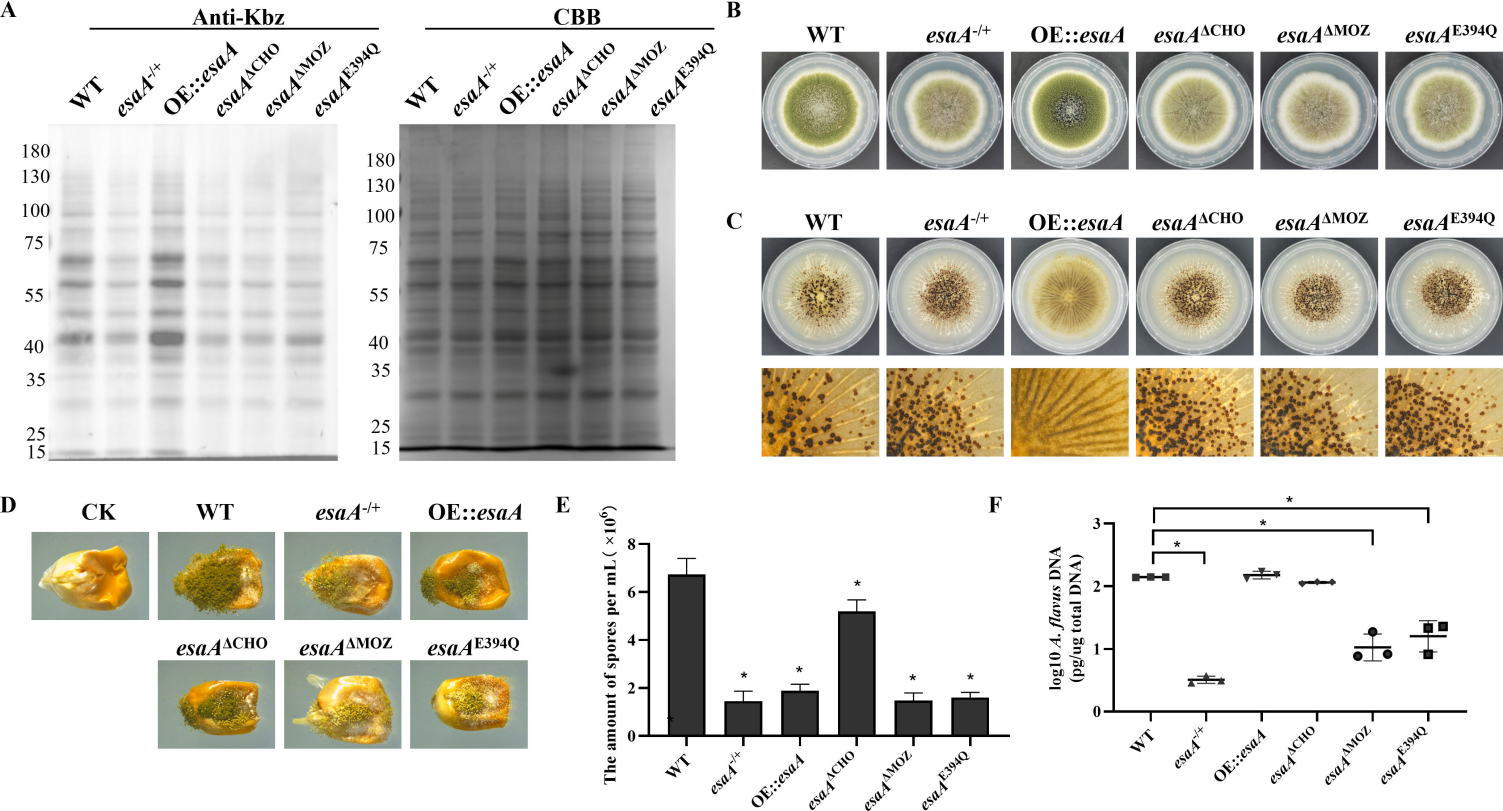
E394 in the MOZ domain is pivotal for EsaA biological function. (**A**) Western blot analysis of Kbz in WT, *esaA*^−/+^, OE::*esaA*, *esaA*^ΔCHO^, *esaA*^ΔMOZ^, and *esaA*^E394Q^ strains. (**B**) Colony morphology of WT and *esaA* mutants grown on YGT media for 5 days. (**C**) Phenotypic characterization of WT and *esaA* mutants grown on CM media for 7 days. (**D**) TLC assay of AFB1 production by the WT and *esaA* mutants (*n* = 3). S indicates AFB1 standard. (**D**) Phenotypic observation of WT and *esaA* mutants in maize kernels. (**E**) Quantification of conidia from the infected maize kernels (*n* = 3). (**F**) Fungal burdens were estimated by quantification of fungal DNA using qPCR (*n* = 3). Asterisks represent statistically significant differences (*P* < 0.05).

EsaA consists of a catalytic domain (monocytic leukemic zinc finger domain, MOZ) and an acyl-lysine binding domain (chromodomain, CHO). To elucidate the function of different domains, the domain deletion mutants *esaA*^ΔCHO^ and *esaA*^ΔMOZ^ were constructed (Fig. S16). The Western blotting results showed that Kbz was decreased in *esaA*^ΔCHO^ and *esaA*^ΔMOZ^ mutants (Fig. 6A), revealing that CHO domain and MOZ domain have important roles in benzoyltransferase activity of EsaA.

EsaA has been proven to play important biological functions in pathogenic fungus (31), but the function of EsaA and its domains was unclear in *A. flavus*, so we examined the role of CHO domain and MOZ domain in vegetative growth, conidiation, sclerotia formation, aflatoxin production, and pathogenicity of *A. flavus*. Phenotypic assays showed that *esaA*^ΔCHO^ and *esaA*^ΔMOZ^ mutants exhibited similar phenotypes to the *esaA*^−/+^ mutant, including reduced colony diameter, fewer conidia, more sclerotia, and AFB_1_ production (Fig. 6B and C; Fig. S17). Meanwhile, overexpression of *esaA* results in decreased sclerotia, increased conidia, and increased AFB_1_ production (Fig. 6B and C; Fig. S17). These data indicate that EsaA and its domain were important for fungal development and aflatoxin synthesis. Moreover, the pathogenic assays showed that all *esaA* mutants produced fewer conidia, fungal burden, and AFB_1_ (Fig. 6D through F; Fig. S18). Taking together, these data demonstrated that CHO domain and MOZ domain of EsaA were crucial for fungal development and pathogenicity in *A. flavus*.

### Benzoyltransferase activity depended on catalytic residue E394 in EsaA

Previous study indicated that E338 (homologous site E394 in *A. flavus*) was a conserved and important catalytic site in EsaA (34). To investigate the function of E394 in EsaA, we generated site mutant *esaA*^E394Q^ (Fig. S16C and D). The Western blotting results showed that Kbz signal was significantly decreased in the *esaA*^E394Q^ mutant (Fig. 6A), suggesting that the E394 site was important for benzoyltransferase activity of EsaA protein. Meanwhile, the *esaA*^E394Q^ mutant displayed a gene deletion and domain deletion mutant-like phenotypes in vegetative growth, conidiation, sclerotia formation, aflatoxin production, and pathogenicity. In detail, when glutamic acid 394 in EsaA protein was mutated to glutamine, fungal growth, conidiation, and pathogenicity were significantly suppressed, while sclerotia formation was remarkably enhanced (Fig. 6B through F; Fig. S17 and S18). All these results revealed that the conserved E394 site was important for biological functions of EsaA.

### Acetylation was important for FoxA biological function in *A. flavus*

**Fig 7:**
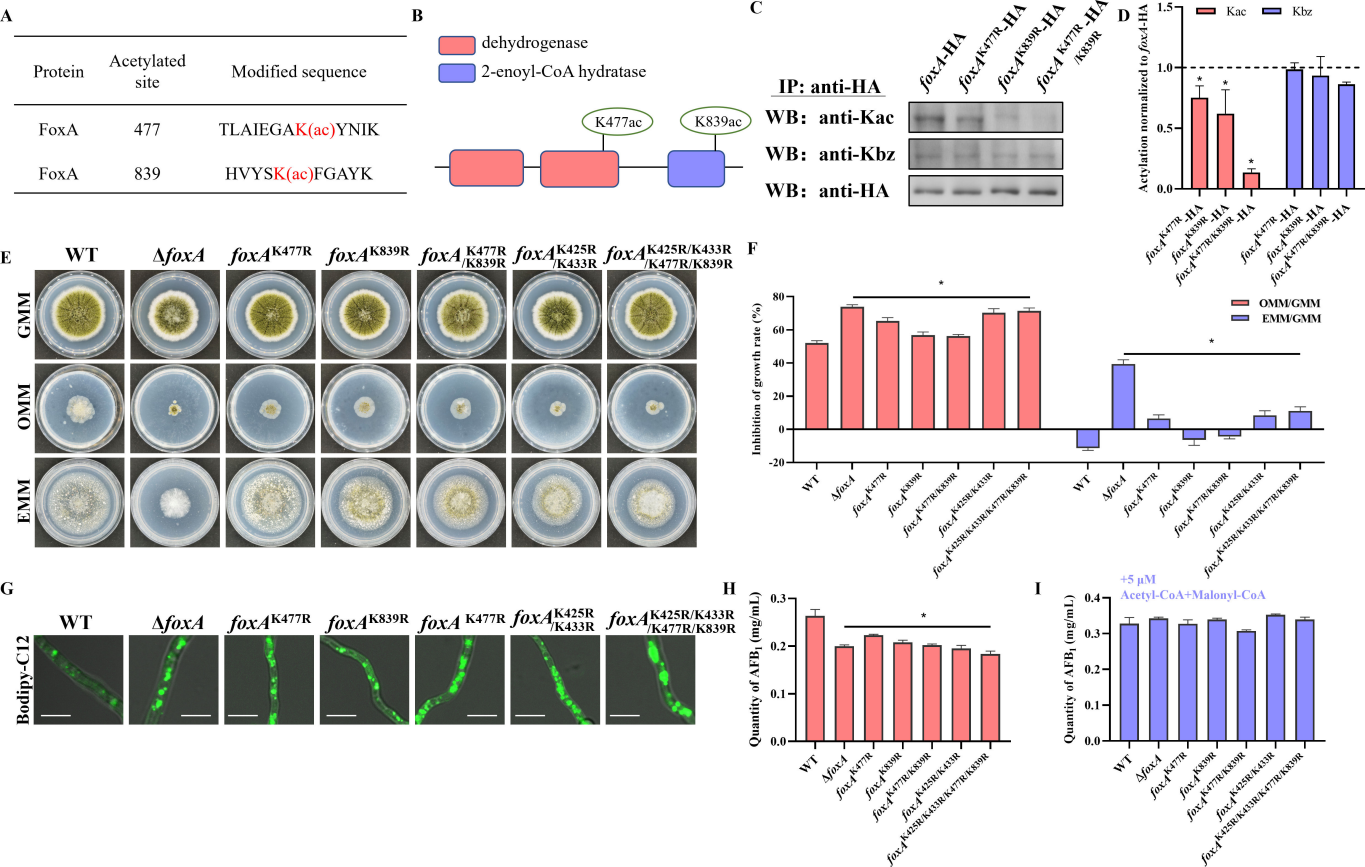
Acetylation also plays an important role in FoxA function. (**A**) Tandem mass spectrometry spectrum results of lysine acetylated sites for FoxA. (**B**) Schematic drawing of the functional domains and acetylated site of FoxA in *A. flavus*. (**C**) Verification of the Kac in FoxA by immunoprecipitation and Western blotting. (**D**) Relative Kbz and Kac levels in *foxA*-HA mutants. (**E**) Colony morphology of WT and *foxA* mutants grown on GMM, OMM, and EMM. (**F**) The inhibition rate of WT and *foxA* mutants (*n* = 3). (**G**) Microscopy of WT and *foxA* mutants stained with BODIPY-C12 for 2 h. Scale bar = 1 µm. (**H**) Quantification of AFB_1_ production in WT and *foxA* mutants (*n* = 3). (**I**) Quantification analysis of AFB_1_ production in WT and *foxA* mutants supplemented with acetyl-CoA and malonyl-CoA (*n* = 3). Asterisks represent statistically significant differences (*P* < 0.05).

Our previous acetylomic results also identified two acetylated sites on FoxA, located at K477 and K839 (Fig. 7A and B). To validate acetylated sites of FoxA, acetylated site mutants (*foxA*^K477R^-HA, *foxA*^K839R^-HA, and *foxA*^K477R/K839R^-HA) were generated (Fig. S19A), in which arginine substitutions were used to mimic unacylated forms of lysine. The WB of the IP product results showed that the Kac signal was reduced in both *foxA*^K477R^-HA and *foxA*^K839R^-HA, with a more pronounced decrease observed in the *foxA*^K477R/K839R^-HA mutant (Fig. 7C and D). Meanwhile, there were no significant differences in Kbz signals among the different FoxA mutants (Fig. 7C and D). In addition, our previous data showed that Kac level of FoxA has no change in OE::*esaA-foxA*-HA, *esaA^−/+^-foxA*-HA, and *gcnE*^xylP^-*foxA*-HA mutants, indicating that *esaA* and gcnE were not responsible for acetylation of FoxA. These results demonstrated that K477 and K839 were indeed acetylated sites in FoxA of *A. flavus*.

To investigate the functions of acetylation in FoxA, we inoculated *foxA*^K477R^, *foxA*^K839R^, and *foxA*^K477R/K839R^ mutants for further study (Fig. S19B). The LCFA utilization results showed that *foxA* deletion and all acetylated site mutants exhibited higher inhibition rates compared to WT (Fig. 7E and F), meaning that defective acetylation of FoxA leads to reduced LCFA utilization activity. Staining with Bodipy-C12 showed that peroxisomes were significantly enlarged in the FoxA acetylated site mutant compared to the WT strain (Fig. 7G), whereas the acetylated site mutation did not disrupt the peroxisomal localization of the FoxA protein (Fig. S20). These results suggested that both K477 and K839 were important regulatory acetylated sites that modulate FoxA function.

To further confirm the impact of acetylation on the biological functional execution of FoxA protein, we detected the role of FoxA acetylation in aflatoxin production, vegetative growth, conidiation, sclerotia formation, and pathogenicity of *A. flavus*. The TLC results showed that all FoxA acetylated site mutants exhibited a reduction in aflatoxin production, similar to the Δ*foxA* strain (Fig. 7H). This reduction could be rescued by the addition of acetyl-CoA and malonyl-CoA (Fig. 7I; Fig. S21). Meanwhile, phenotypic data showed that all FoxA acetylated site mutants (*foxA*^K477R^, *foxA*^K839R^, *foxA*^K477R/K839R^) exhibited similar phenotypes to Δ*foxA* mutant, including reduced colony diameter, fewer conidia, and more sclerotia (Fig. S22). Meanwhile, the pathogenic assays showed that all FoxA mutants exhibited lower pathogenicity with decreased fungal DNA burden, fewer conidia, and less AFB_1_ (Fig. S23). Taken together, these data demonstrated that acetylation of FoxA was important for its biological function in *A. flavus*.

### Both benzoylation and acetylation were important in FoxA protein

Our findings indicated that benzoylation and acetylation play critical roles in the functional execution of FoxA proteins, and mutations at either the benzoylated or acetylated sites do not alter the levels of another modification. To further investigate the relationship between benzoylation and acetylation of FoxA, we constructed *foxA*^K425R/K433R/K477R/K839R^ mutants (Fig. S19B). The LCFA utilization results showed that four acylated site mutants exhibited a similar inhibition rate to benzoylated site mutant *foxA*^K425R/K433R^ and acetylated site mutant *foxA*^K477R/K839R^ (Fig. 7E and F). Moreover, peroxisomes in all four acylated sites mutants were markedly enlarged (Fig. 7G), although the FoxA localization remained unaffected (Fig. S20). These results indicating both benzoylation and acetylation were important in the function of FoxA protein, and they might regulate FoxA activity in a similar and independent way. Meanwhile, the phenotypic data revealed that all acylated site mutants (*foxA*^K425R/K433R/K477R/K839R^) exhibited similar phenotypes to *foxA*^K425R/K433R^ mutant and *foxA*^K477R/K839R^ mutant, including decreased colony diameter, fewer conidia, lower AFB_1_ production, weak pathogenicity, and increased sclerotia (Fig. 7H and I; Fig. S22 and S23). Taking together, benzoylation and acetylation at different sites on the FoxA protein modulate the development and virulence of *A. flavus* by influencing the functional execution of FoxA.

## DISCUSSION

Fatty acid β-oxidation is a process by which fatty acids break down to acetyl coenzyme A and generate ATP to ensure cellular energy supply. FoxA, a multifunctional enzyme encoding trans-2-enoyl CoA hydratase and 3-hydroxyacyl CoA dehydrogenase, can catalyze the second step of hydration and the third step of dehydrogenation during peroxisomal β-oxidation (27). The acylation proteome revealed the presence of benzoylation and acetylation in FoxA protein, which were successfully verified by immunoprecipitation and immunoblotting experiments. Combined with long-chain fatty acid utilization experiments and metabolomic data, the important role of FoxA protein in peroxisomal β-oxidation was confirmed, and phenotyping experiments clarified the effects of benzoylation and acetylation on FoxA protein function in *A. flavus*.

In filamentous fungi, β-oxidation mainly occurred in peroxisomes and mitochondria. Long- and ultra-long chain fatty acids are mainly shortened by the peroxisomal β-oxidation pathway, whereas short- and medium-chain fatty acids are mainly degraded by the mitochondrial β-oxidation pathway (26, 35–38). The mechanism of fatty acid degradation in mammals was similar to filamentous fungi (38). Consistent with mitochondrial acyl-CoA dehydrogenase *scdA* and mitochondrial enoyl-CoA hydratase *echA* double deletion mutant (Δ*scdA*/*echA*), the mitochondrial ketoacyl-CoA thiolase *mthA25* deletion strain exhibited severe growth defects on short-chain fatty acids, but has no effect on long-chain fatty acids (35, 36). In contrast, disruption of the *foxA* gene prevented the use of long and ultra-long chain fatty acids as carbon sources, but no effect on short- and medium-chain fatty acids in *A. nidulans* (26). In this study, deletion of the *foxA* gene in *A. flavus* not only hinders fungal growth on oleic acid (C18) and erucic acid (C22) (Fig. 1D and E), but also impairs fatty acid β-oxidation so fungus cannot efficiently degrade the long-chain fatty acids, including 2-arachidonic acid glycerol, α-linolenic acid, dodecanedioic acid, hypoglyceridic acid, γ-linolenic acid methyl ester, and octadecanoic acid (Fig. 3C). In addition, metabolomic data also showed that deletion of the *foxA* gene could affect lipid metabolism, amino acids, and carbohydrate metabolisms in *A. flavus*, and qRT-PCR results indicated that the loss of FoxA function leads to the upregulation of various metabolic synthases (Fig. 3F). This upregulation likely occurs as a compensatory response to the blocked degradation of long-chain fatty acids and the resulting metabolic flux change. Collectively, these findings revealed the pivotal role of the FoxA protein in the broader metabolic network of *A. flavus*. However, for *Saccharomyces cerevisiae* and *Candida albicans*, β-oxidation occurs only in peroxisomes (39, 40), and β-oxidation was enhanced when the fungus interacts with the host or nutritionally depleted (41). Previous studies have shown that FoxA protein was involved in regulating the degradation of aromatic compounds, and *foxA* gene deletion leads to a significant decrease in the utilization of hydroxycinnamic acid in *Aspergillus niger* (24). These results illustrated the functional diversity of FoxA protein in fungi.

FoxA protein was involved in fungal growth and development. Deletion of the *foxA* gene resulted in decreased conidium production and increased sclerotium production by regulating core transcriptional factors *abaA*, *wetA,* and *nsdC*, respectively. Although fungi have the ability to synthesize their own fatty acid, the majority of fatty acid that enters into the β-oxidation cycle is exogenous fatty acid, and the produced energy can be utilized to maintain the fungal development (37). Fatty acid metabolism plays an important role in fungal sexual reproduction (38). In *Aspergillus nidulans*, the balance between sexual and asexual development was mainly controlled by the oxidized lipids psiBβ and psiBα, which were derived from oleic and linoleic acids, respectively (42). Peroxisome was required at a specific stage of sexual development in *Podospora anserina* (43). These results implied that the FoxA may also regulate fungal development by affecting the formation of oxidized lipids. The metabolism results showed that *foxA* gene deletion caused an increased production of 6-gingerol, nogalamycin, and 15d-PGJ(2)-G. It has been reported that 6-gingerol could induce apoptosis (44), while nogalamycin could inhibit DNA replication (45). Our results also showed that 15d-PGJ(2)-G could suppress the growth and conidiation of *A. flavus*. Consequently, FoxA could regulate fungal development by affecting secondary metabolism in *A. flavus*.

Fatty acid β-oxidation is one of the main suppliers of intracellular acyl-CoA, which is not only a core substance of primary metabolism but also participates in secondary metabolism. Fatty acids not only act as transcriptional activators to induce aflatoxin synthesis, but also stimulate cells to produce large amounts of acyl-CoA, which provides raw material acyl-CoA and malonyl coenzyme A for aflatoxin synthesis (13). Compared with low-oleic peanuts, high-oleic peanuts were liable to produce more aflatoxins under *Aspergillus* contamination (19). Oleic acid as a peroxisome proliferator induces a rise in the expression of the *foxA* gene and increases the intracellular acyl-CoA production in *Aspergillus*, which, in turn, promotes the synthesis of sterigmatocystin and aflatoxins (20). TLC and metabolomic results indicated that deletion of the *foxA* gene resulted in downregulated aflatoxin cluster genes and decreased aflatoxin production. We speculate that there were two reasons for reduced aflatoxin. On the one hand, the *foxA* gene was closely related to the acyl-CoA formation, and its deletion caused decreased content of acyl-CoA, eventually leading to insufficient raw materials for aflatoxin synthesis. For example, overexpression of fox2 was able to enhance the acyl-CoA production in *Saccharomyces cerevisiae* (17), and peroxisome proliferators can promote aflatoxin synthesis by enhancing the expression of the *foxA* gene (21). On the other hand, deletion of the *foxA* gene affects aflatoxin synthesis by reducing peroxisomal transport. Deletion of the *fox2* gene leads to enlarged and mutated peroxisomes in *C. albicans* and *C. lusitaniae* (28, 46), resulting in a reduced membrane volume ratio and, ultimately, a decreased transport capacity of peroxisomes. Overproduction of *pex11* resulted in enhanced penicillin and tubular peroxisomes proliferation, while the transcriptional level of penicillin synthesis cluster gene remained unchanged in *Penicillium chrysogenum* (47), suggesting that an increased membrane volume ratio may enhance fungal secondary metabolism by improving peroxisomal transport. In *A. flavus*, deficiency in FoxA leads to an elevated level of 15d-PGJ(2)-G, which induces abnormal peroxisomal enlargement. This morphological alteration results in a diminished transport capacity and a subsequent reduction in aflatoxin production.

Our founding suggested that FoxA influences fungal colonization and pathogenicity in *A. flavus*. This is consistent with previous studies that *mfe2* (*foxA* orthology) mutants exhibited significantly reduced virulence and tumor production in *Ustilago maydis* (48). Moreover, fatty acid β-oxidation has been shown to play an important role in fungal pathogenicity. Deletion of the *pex6* gene leads to loss of peroxisomal metabolic function and triggers defective appressorial penetration in *Colletotrichum lagenarium* (47). Combined with the metabolomic data, we hypothesized that the absence of the *foxA* gene resulted in impaired fatty acid β-oxidation and an inability to efficiently utilize the LCFAs in the maize kernel, thereby preventing the proper utilization of host nutrients and slowing down the fungal proliferation in the host.

Both K425 and K433 have been recognized as important regulatory benzoylated sites within FoxA protein, which is supported by abolished Kbz signal of FoxA in double site mutants. Like other fungi, FoxA protein has three conserved domains: two dehydrogenase domains and a 2-enoyl-CoA hydratase domain (29). We found that benzoylated sites appeared in the dehydrogenase domain. Given that the benzoylated sites appeared in the dehydrogenase domain, we imply that benzoylation of FoxA was tightly related to dehydrogenase activity. As expected, the long-chain fatty acid utilization and phenotype of the benzoylated point mutants (K425R, K433R, and K425R/K433R) showed a strong similarity to that of the gene deletion mutant, including reduced LCFA utilization activity, abnormal enlargement of peroxisome, lower seed colonization, increased sclerotia formation, and decreased conidiation and aflatoxin production. Compared to *foxA*^K433R^, *foxA*^K425R^ displays a stronger defective LCFAs utilization, indicating that benzoylated K425 site plays a major role in regulating FoxA function. The lack of further enhancement in the double mutant indicates that benzoylated sites may function in a non-redundant manner within a common functional pathway or structural module. Moreover, mutations in the benzoylated sites had no effect on FoxA protein localization. These results suggested that defective benzoylation of the FoxA protein leads to reduced activity of FoxA, and two benzoylated sites were required for the functional execution, demonstrating that benzoylation has a critical role in the functional execution of the FoxA protein.

The identified acetylation sites K477 and K839 were located within the dehydrogenase domain and the 2-enoyl-CoA hydratase domain, respectively. Consistent with benzoylated site mutants, acetylated site mutants also exhibited defective FoxA function and decreased aflatoxin production, akin to the *foxA* gene deletion mutant. These findings indicate that acetylation plays a crucial role in the functional performance of the FoxA protein. Interestingly, although both the benzoylation and acetylation sites are situated within the enzymatic domains, none of these four lysine residues correspond to the core catalytic residues of the FoxA protein (Fig. S24). Previous studies in *Arabidopsis* have established that the catalytic activity relies on specific key motifs, with the critical active sites identified as Glu^119^ and Glu^139^ in the enoyl-CoA hydratase domain and a conserved NAD^+^-binding motif (^319^Gly-X-Gly^321^-X-X-Gly^324^) in the dehydrogenase domain, both essential for enzymatic function (49). This suggested that the substitution of these lysine residues with arginine (KR mutations) does not directly disrupt the enzyme’s active site. Instead, the loss of these specific acyl modifications likely impairs FoxA function through alternative mechanisms, such as by affecting protein stability, conformational dynamics, or interactions with other regulatory partners. A comparative analysis of the impacts of acetylation and/or benzoylation defects on FoxA functionality and phenotype demonstrated a more similar phenotypic and enzymatic characterization between the benzoylated site mutant (*foxA*^K425R/K433R^) and the acylated site mutant (*foxA*^K425R/K433R/K477R/K839R^). Meanwhile, the Western blot analysis indicated that mutations at the Kbz sites of the FoxA protein did not influence its acetylation levels, nor did mutations at the Kac sites affect its benzoylation levels. This suggested that Kbz and Kac regulate the function of FoxA in an independent manner. Numerous instances of crosstalk among various modifications have been documented (8), and the effects on the proteins are not always the same. Acetylation and ubiquitination of RelA protein were inhibited by each other with their use of overlapping sites (50). Glycosylation of threonine 19 on ENO1 promotes its glycolytic activity (51), whereas decreased 2-hydroxyisobutyrylation at lysine 281 and reduced crotonylation levels at lysine 420 inhibit its enzymatic activity (52, 53). Taken together, complex post-translation modifications may coordinately or independently regulate various biological processes.

It has not been previously reported that FoxA undergoes post-translational modification, so this study is the first time to prove that FoxA protein has both benzoylation and acetylation in *Aspergillus flavus*. We found that *esaA* expression positively regulates FoxA benzoylation and LCFA utilization without affecting acetylation, demonstrating that EsaA acts as the specific benzoyltransferase for FoxA and is essential for fatty acid β-oxidation. However, the repression of *gcnE* has no effect on benzoylation and acetylation of FoxA, revealing that there were other acyltransferases responsible for the FoxA acetylation in *A. flavus*. Numerous studies have discovered that histone acyltransferase EsaA exhibits activities for acetylation, crotonylation, 2-hydroxyisobutyrylation, and histone benzoylation (2, 7, 31, 54). EsaA could affect the functional execution of FoxA by catalyzing its benzoylation. Similarly, it could mediate the functional execution of other acylation-modified proteins. For example, the acetyltransferase Esa1 promotes autophagy by acetylating ATG3 in *S. cerevisiae* (55). The Esa1 protein enhances the acetylation of the Pif1 protein, thereby activating its helicase, ATPase, and DNA-binding activities in *S. cerevisiae* (56). Therefore, EsaA could modulate various biological processes by modifying different substrate proteins.

## MATERIALS AND METHODS

### Strains and culture conditions

The *A. flavus* strains used in this study were listed in Table S1. All strains were cultured on YGT agar medium or GMM agar medium for growth and conidiation assays. Then, conidia were harvested and counted using a hemocytometer and under a microscope (57). Sclerotia were harvested from CM agar medium, and aflatoxins were extracted from YES or GMM liquid medium (58). To explore fatty acid metabolism, oleic acid (6 mM) or erucic acid (4.9 mM) was substituted for glucose in GMM medium (26).

### Construction of mutant strains

The gene deletion mutant (Δ*foxA*) and transformation of *A. flavus* were carried out using the protocols described previously (59). Overlap PCR was performed to fuse the target gene upstream sequence, downstream sequence, and selectable marker (*pyrG*). Then, the fusion products were transformed into CA14 protoplasts of *A. flavus*. The complementation mutant (*foxA*-com) was constructed using a previously described protocol (60), and 5-fluoroorotic acid (5-FoA) was used to screen the *pyrG* deletion mutant (61). Site mutants (*foxA*^K425R^, *foxA*^K433R^, *foxA*^K425R/K433R^, *foxA*^K477R^, *foxA*^K839R^, *foxA*^K477R/K839R^, and *foxA*^K425R/K433R/K477R/K839R^) and HA-tag fused mutants (*foxA*-HA, *foxA*^K425R^-HA, *foxA*^K433R^-HA, *foxA*^K425R/K433R^-HA, *foxA*^K477R^-HA, *foxA*^K839R^-HA, *foxA*^K477R/K839R^-HA, OE::*esaA-foxA*-HA, *esaA^−/+^-foxA*-HA, and *gcnE*^xylP^-*foxA*-HA) were constructed through the fixed-point mutation primers (62). PCR and RT-PCR were used to confirm the positive transformants. All primers used for mutant construction and verification were listed in Table S2.

### QPCR assays

Total RNA was isolated by TRIzol reagent, and cDNA was synthesized with 1st Strand cDNA Synthesis Kit (Vazyme, R312-01). The qPCR assay was performed on Real-time PCR PikoReal 96 system using qPCR SYBR Green Master Mix (Yeasen, 11201ES08). All qPCR primers were listed in Table S3. Relative quantification was determined by standard curve method, and *actin* gene was used as a housekeeping gene for normalization (57). Each experiment was repeated three times.

### Validation of acylated proteins and sites *in vivo*

We mutated lysine to arginine and confirmed HA-tag fusion mutants by sequencing and WB. Anti-HA magnetic beads (MCE, HY-K0201) were used for IP experiments, and the bound proteins were eluted by protein loading buffer. Then, anti-HA antibody (CST, 3724S), pan anti-benzoylation (anti-Kbz) antibody (PTM Biolabs, PTM-762), and pan anti-acetylation (anti-Kac) antibody (PTM Biolabs, PTM-102) were used for immunoblotting, respectively.

### Western blot

The protein was extracted using RIPA lysis buffer (Beyotime, P0013B), separated by SDS-PAGE, and then transferred to PVDF membranes (Millipore, IPVH00010). Subsequent transformants were subjected to Western blotting analysis with pan anti-Kbz, pan anti-Kac, and anti-HA primary antibodies and anti-rabbit and anti-mouse secondary antibodies (Thermo, 31460 and 31430). Then, the proteins were detected using an Ultra High Sensitivity ECL Kit (Glpbio, GK10008) in GBox XT4 Chemiluminescence and Fluorescence Imaging System.

#### BODIPY-C12 staining

Fresh spore suspension was inoculated into OMM liquid medium and cultured overnight at 37°C. The cells were then harvested by centrifugation at 12,000 r/min, and the supernatant was discarded. Then, the pellet was washed three times with PBS. Subsequently, 5 µM BODIPY 500/510 C1, C12 dye (Beyotime, C2055) was added, and the samples were incubated in the dark at 37°C for 2 h. After incubation, the cells were washed three times with PBS again and observed under a fluorescence microscope.

#### Fluorescence detection

Fresh spores were inoculated into liquid medium and cultured overnight at 37°C. The supernatant was discarded, and the cells were washed with PBS to remove the medium. The cells were fixed with 4% paraformaldehyde at room temperature for 20 min. After removing the paraformaldehyde solution, the cells were washed three times with PBS. Permeabilization was performed using PBS containing 0.5% Triton X-100 at room temperature for 30 min, followed by three 5-minute washes with PBS. Blocking was carried out with PBS containing 1% BSA at room temperature for 30 min. The cells were then incubated with HA antibody solution (diluted 1:100 in PBS with 1% BSA) at 4°C overnight. Following incubation, the cells were washed three times with PBS for 10 min each. Subsequently, the cells were incubated with a mixture of anti-rabbit antibody (Alexa Fluor 594 Conjugate, Servicebio, GB28301) and BODIPY-C12 solution in PBS containing 1% BSA, protected from light at room temperature for 2 h. Finally, the cells were washed three times in the dark with PBS for 5 min each. Hyphae were visualized under a fluorescence microscope.

### Phenotypic assays

For analysis of conidia and sclerotia formation, a 10^3^ conidia suspension was inoculated onto the plates of YGT, GMM, and CM medium. Colony morphology was observed with strains grown on YGT or GMM medium at 37°C for 5 days, and colony diameters and conidia were measured and counted. Sclerotia were harvested from CM agar medium after being incubated in the dark at 37°C for 7 days. Mycelia and conidia were removed using 75% ethanol, and the sclerotia were then counted under anatomical lens (60). Each experiment was performed three times with three replicates.

### Aflatoxin assays

For aflatoxin assay, a suspension of 10^4^ conidia was cultured on YES or GMM liquid medium in the dark at 29°C for 7 days. Then, aflatoxin was extracted by dichloromethane as described earlier (57). Aflatoxin B_1_ was detected and quantified by thin-layer chromatography (TLC). Each experiment was performed with three replicates three times.

### Infection assays

The infection assay was performed according to previously reported methods (58). Viable maize seeds were sterilized, washed, and inoculated with spores, then cultured in the dark at 29°C. Meanwhile, maize seeds were inoculated with sterilized water for control. After maize seeds were harvested, conidiophores, aflatoxin production, and fungal burden were quantified at 5 dpi.

### *In vitro* benzoylation assays

The AflEsaA full-length DNA was cloned into pET-32a and transformed into the *E. coli* Rosetta (DE3) strain. Protein expression was induced with IPTG, and cell pellets were broken by ultrasound (63). His-tag fusion protein was eluted with a gradient of imidazole buffer, with the finally eluted concentration of imidazole being 300 mM. *In vitro* benzoylation assay was performed based on previously reported method (54, 64).

### Non-targeted metabolomics assays

Fresh conidia of WT, Δ*foxA,* and *foxA*^K425R/433R^ strains were inoculated into YES liquid medium and incubated at 29°C with shaking at 180 r/min. The mycelia were then harvested and sent to Zhongke New Life Biotechnology Co., Ltd. (Shanghai, China) for non-targeted metabolomics. UHPLC-MS-based metabolomic analysis and sample extraction were performed as described previously (18, 65). The *P*-values were adjusted using the Benjamini-Hochberg false discovery rate (FDR) correction method. Ions with an FDR < 0.01 and a log2 fold change (FC) > 2 or < −2 were deemed to be significantly differentially expressed (66).

### Statistical analysis

Data are presented as the means ± standard deviation (SD) from at least three biological replicates. The data were processed by GraphPad Prism 8.0 and SPSS 19.0. Significance was evaluated by one-way analysis of variance (ANOVA) using LSD test, and *P*-value < 0.05 was considered to indicate a significant difference.

## Data Availability

All the raw data of the metabolome were uploaded to the China National Gene Bank database with the identifier CNP0006675.

## SUPPLEMENTAL MATERIAL

Supplemental material mbio.03672-25-s0001.docx Fig. S1 to S24; Tables S1 to S3.

ASM does not own the copyrights to Supplemental Material that may be linked to, or accessed through, an article. The authors have granted ASM a non-exclusive, world-wide license to publish the Supplemental Material files. Please contact the corresponding author directly for reuse.
