# Supplementary material for: Lysine benzoylation of fatty acid β-oxidation core enzyme FoxA regulates the aflatoxin biosynthesis by benzoyltransferase EsaA in the pathogenic fungus Aspergillus flavus
Source: mBio. 2026 Apr 20;17(5):e03672-25. doi: 10.1128/mbio.03672-25 (PMC13170224; doi:10.1128/mbio.03672-25)
Supplement: Supplemental material — Fig. S1 to S24; Tables S1 to S3. [file mbio.03672-25-s0001.docx]

**Supplemental material**

**Fig S1. The benzoylated sites of FoxA.**

(A) The benzoylated peptide VTK(benzoyl)-AAWPYMLK (benzoyl)-QK with the crotonylation site at K321 in the FoxA. (B) Tandem mass spectrometry spectrum results of lysine benzoylated sites for FoxA. (C) Conserved site analysis of benzoylated peptited in FoxA protein.


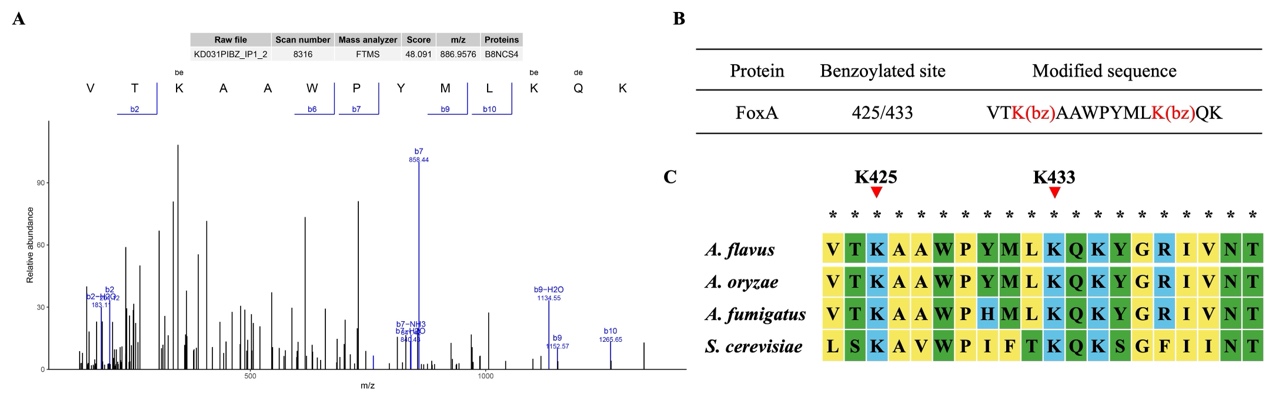


**Fig S2. *foxA* plays important role in fungal development.**

(A) Construction strategies of Δ*foxA* and *foxA*-com strains. (B) Genomic PCR (B) and qPCR (C) verification of Δ*foxA* and *foxA*-com strains. (D) TLC assay of AFB_1_ production by the WT, Δ*foxA* and *foxA*-com cultured in GMM liquid media at 29℃ for 7 days. S indicates AFB_1_ standard. (E) Quantification analysis of AFB_1_ in TLC results by optical density (n=3). (F) Colony morphology of WT, Δ*foxA* and *foxA*-com strains grown on YGT media for 5 days. (G) Amount of conidia produced by the different strains on YGT media (n=3). (H) Phenotypic characterization of WT, Δ*foxA* and *foxA*-com strains grown on CM media for 7 days. (I) Amount of sclerotia produced by different strains (n=3). (J) Relative expression level of *abaA* and *wetA* genes (n=3). (K) Relative expression level of *nsdC* gene (n=3). Asterisks represent statistically significant differences (P<0.05).


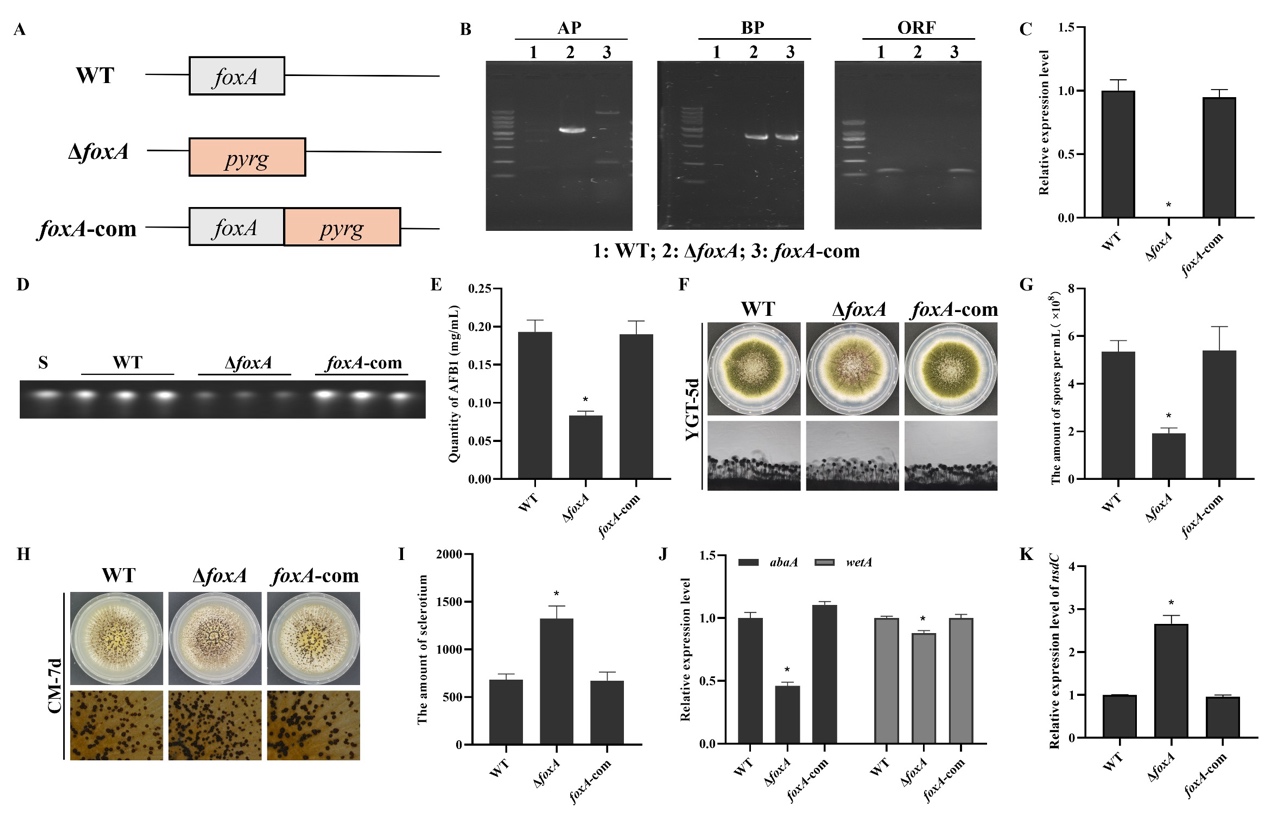


**Fig S3. Construction and confirmation of *foxA*-HA and *foxA* mutants.**

(A) Sequencing confirmation of *foxA*-HA mutants. (B) Sequencing confirmation of benzoylated site mutants.


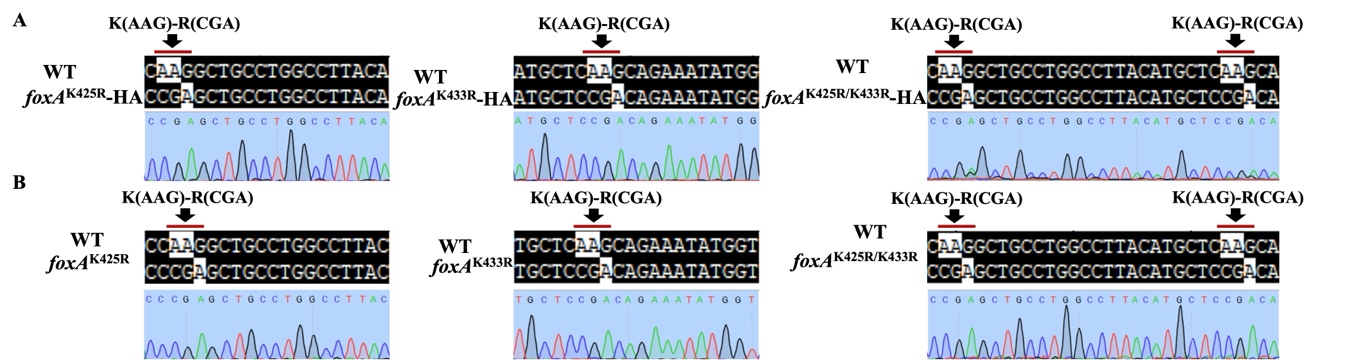


**Fig S4. Localization analysis of FoxA protein in *foxA*-HA and *foxA*^K425R/433R^-HA.**

**
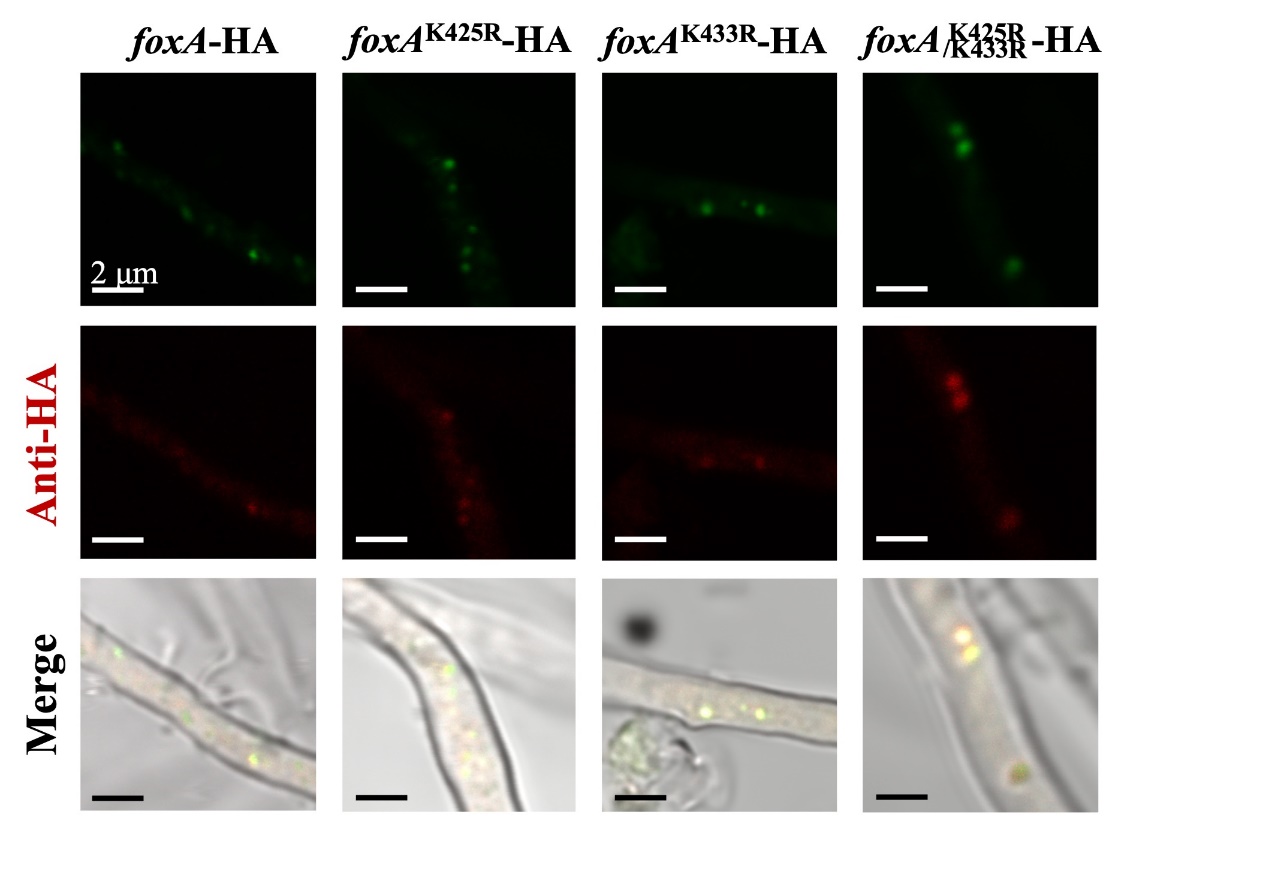
**

**Fig S5. The addition of acetyl-CoA and malonyl-CoA induces an increase in aflatoxin synthesis.**

(A) TLC analysis of AFB_1_ production in the WT strain treated with different concentration of acetyl-CoA and malonyl-CoA (n=3). S indicates AFB_1_ standard. (B) Quantification analysis of AFB_1_ in TLC results by optical density (n=3). Asterisks represent statistically significant differences (P<0.05).

**
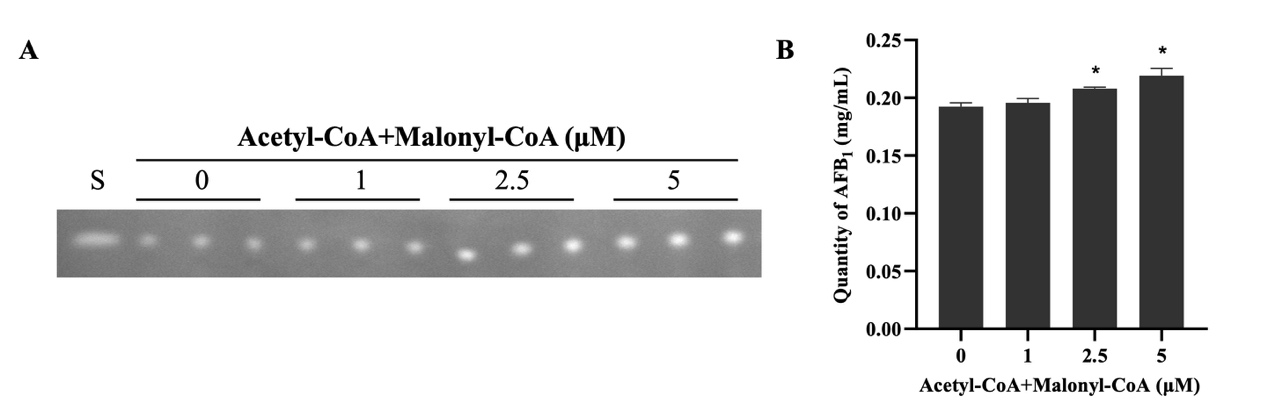
**

**Fig S6. The Kbz of FoxA play important roles in crop colonization.**

(A) Phenotypic observation of WT and *foxA* mutants in maize kernels. (B) Maize kernels were harvested, and fungal burdens were estimated by quantification of fungal DNA using qPCR (n=3). Asterisks represent statistically significant differences (P<0.05).

**
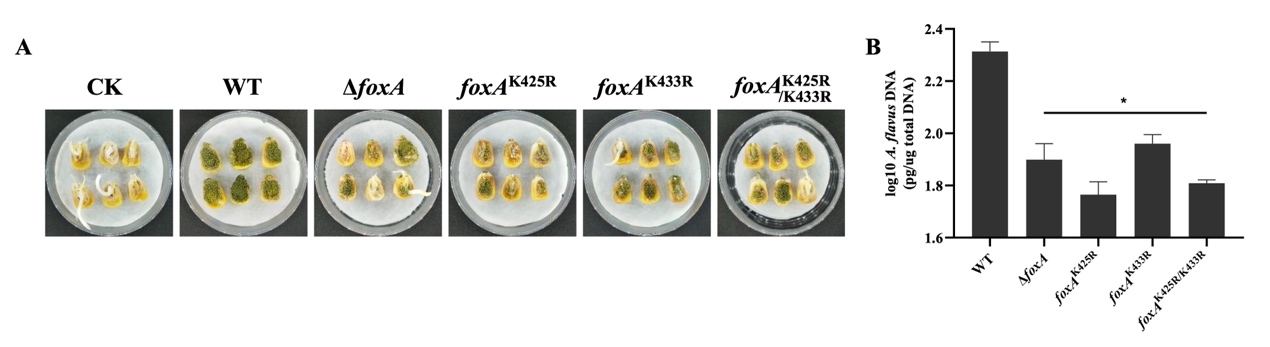
**

**Fig S7. The Kbz of FoxA was required for fungal development.**

(A) Colony morphology of WT and *foxA* mutants grown on GMM media at 37℃ for 5 days. (B) Phenotype of WT and *foxA* mutants on CM media, after growing for 7 days at 37°C. (C-D) Colony diameter (C) and conidia production (D) by different strains on GMM media (n=3). (E) Amount of sclerotia produced by different strains on CM media (n=3). Asterisks represent statistically significant differences (P<0.05).


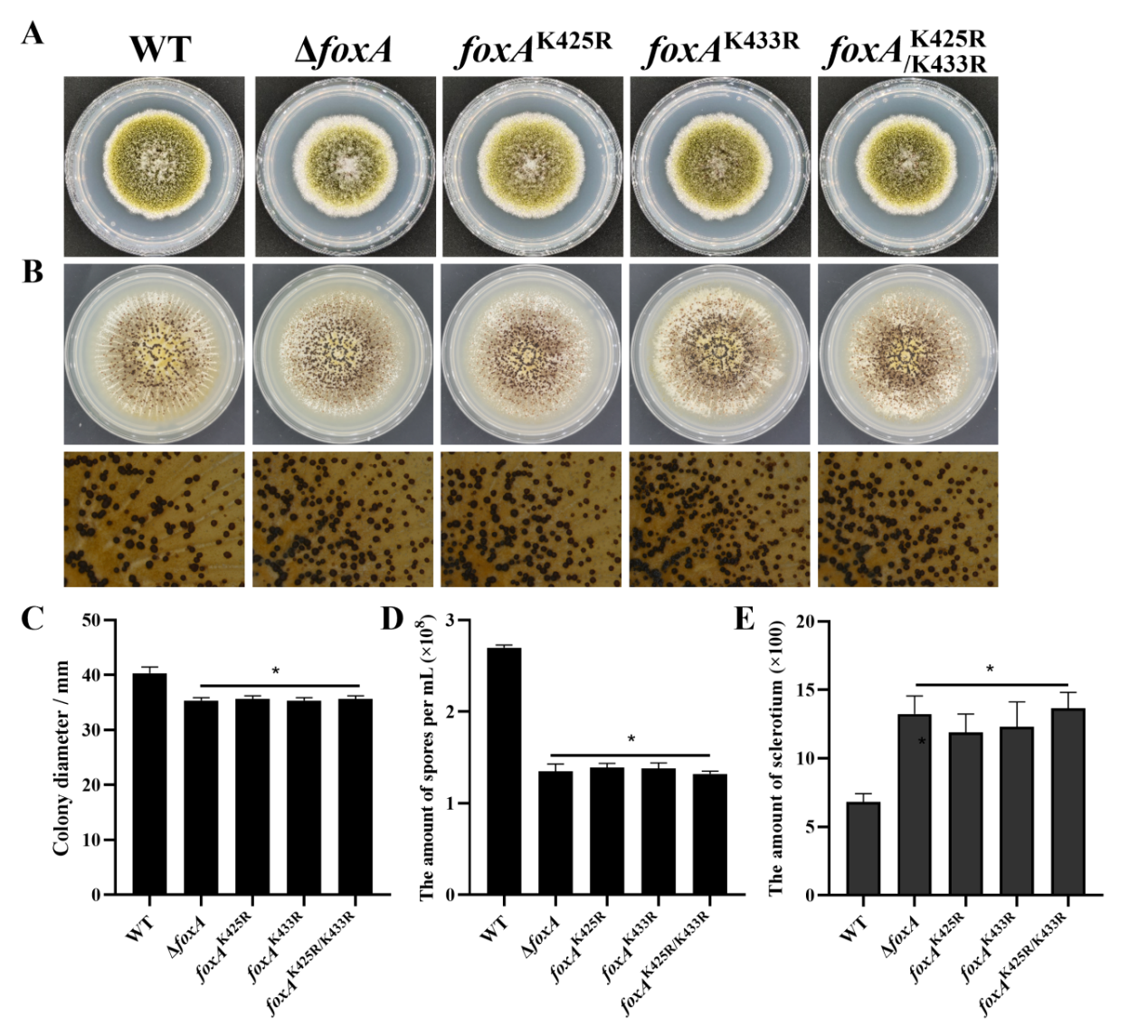


**Fig S8. Metabolomic analysis of WT, Δ*foxA* and *foxA*^K425R/K433R^ strains.**

(A) Total ion chromatogram of QC sample in positive ion mode. (B) Total ion chromatogram of QC sample in negative ion mode. (C) PCA analysis. (D) Classification of metabolites.


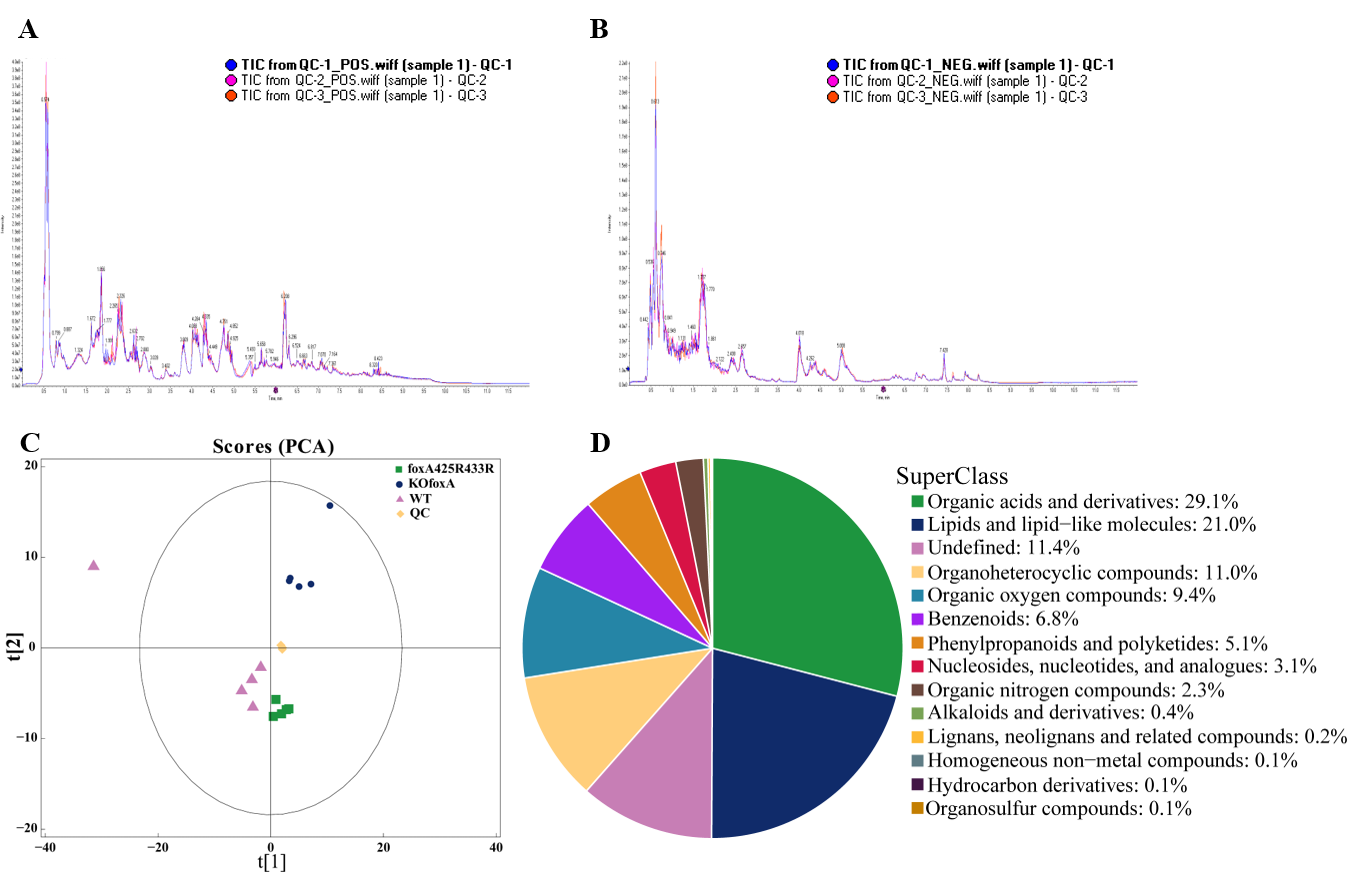


**Fig S9. Differential metabolites analysis.**

(A) Differential metabolites of WT and Δ*foxA* strains in the positive ion mode. (B) Differential metabolites of WT and Δ*foxA* strains in the negative ion mode. (C) Differential metabolites of WT and *foxA*^425R/433R^ strains in the positive ion mode. (D) Differential metabolites of WT and *foxA*^425R/433R^ strains in the negative ion mode. (E) Venn plot of positive ion mode. (F) Venn plot of negative ion mode.


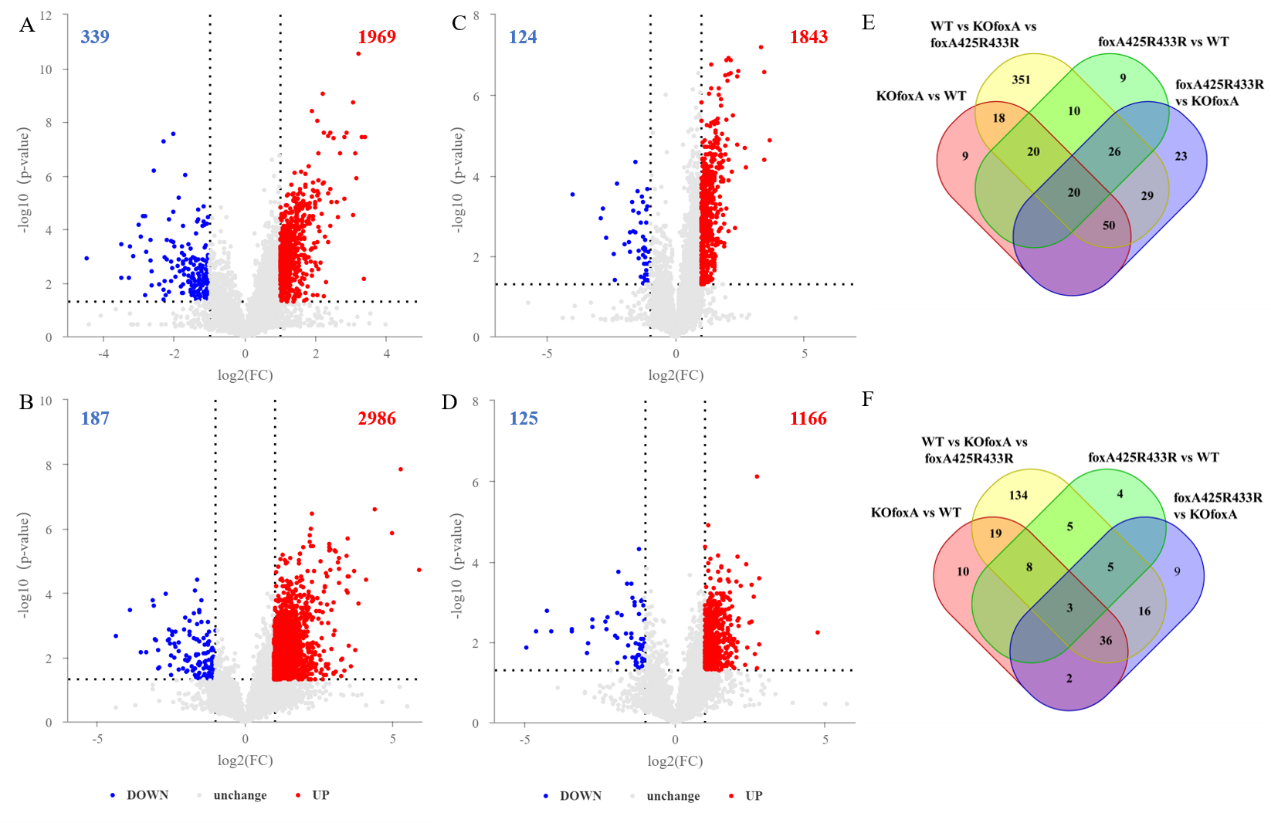


**Fig S10. The KEGG analysis of differential metabolites.**

(A-B) Differential abundance score spot of differential metabolites in Δ*foxA* (A) and *foxA*^425R/433R^ (B) strains compared to WT. (C) The heatmap of differential metabolites in the ABC transporter pathway (ko02010). (D) The heatmap of differential metabolites in the biosynthesis of amino acids (ko01230).


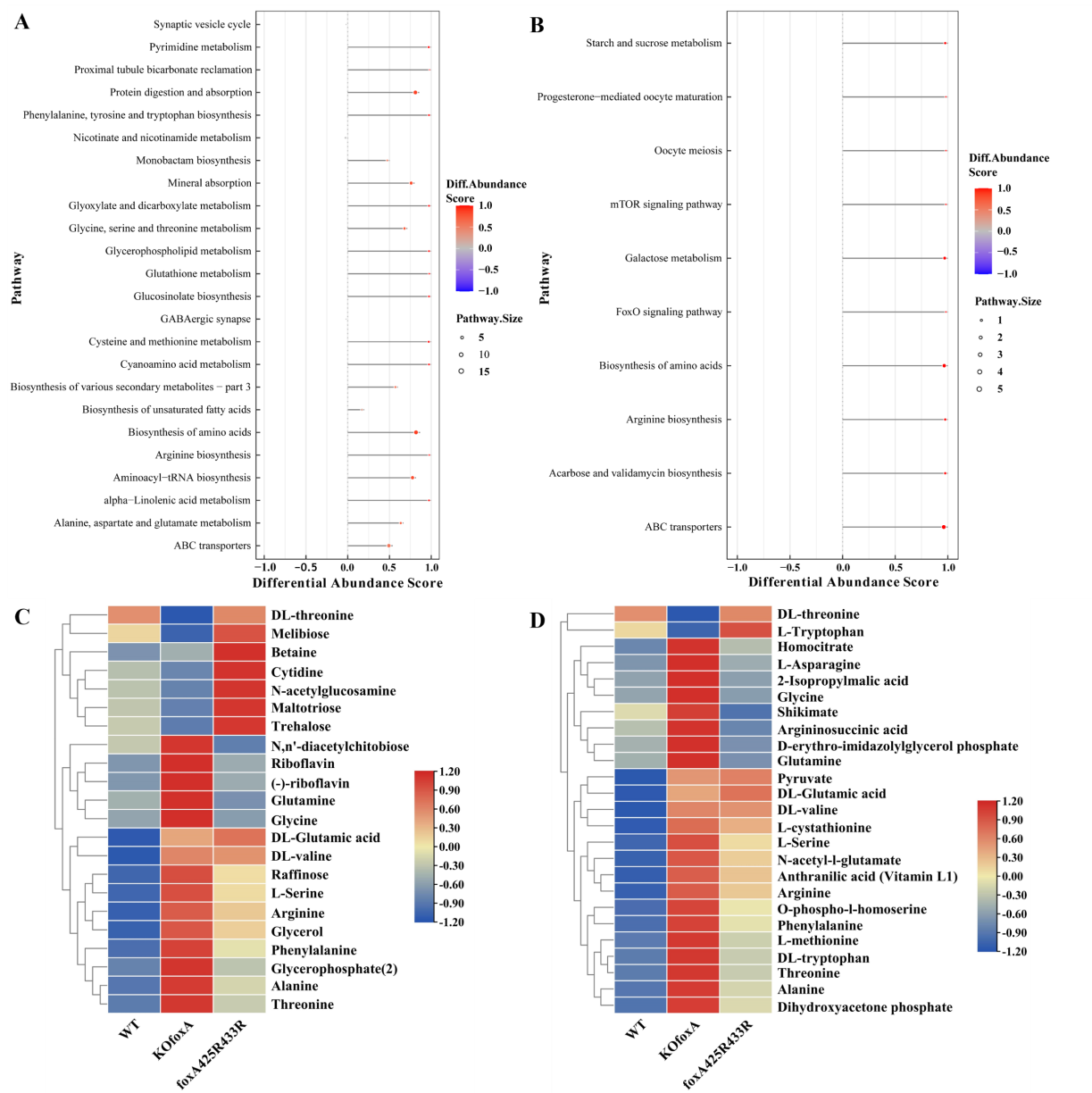


**Fig S11. Construction and confirmation of *esaA* and *gcnE* mutants.**

(A) Estimation of copy number of *esaA* gene in *A. flavus*. (B) Schematic diagram of *esaA* mutants. (C-D) Genomic PCR verification of *esaA*^-/+^(C), OE::*esaA* (D) strains.


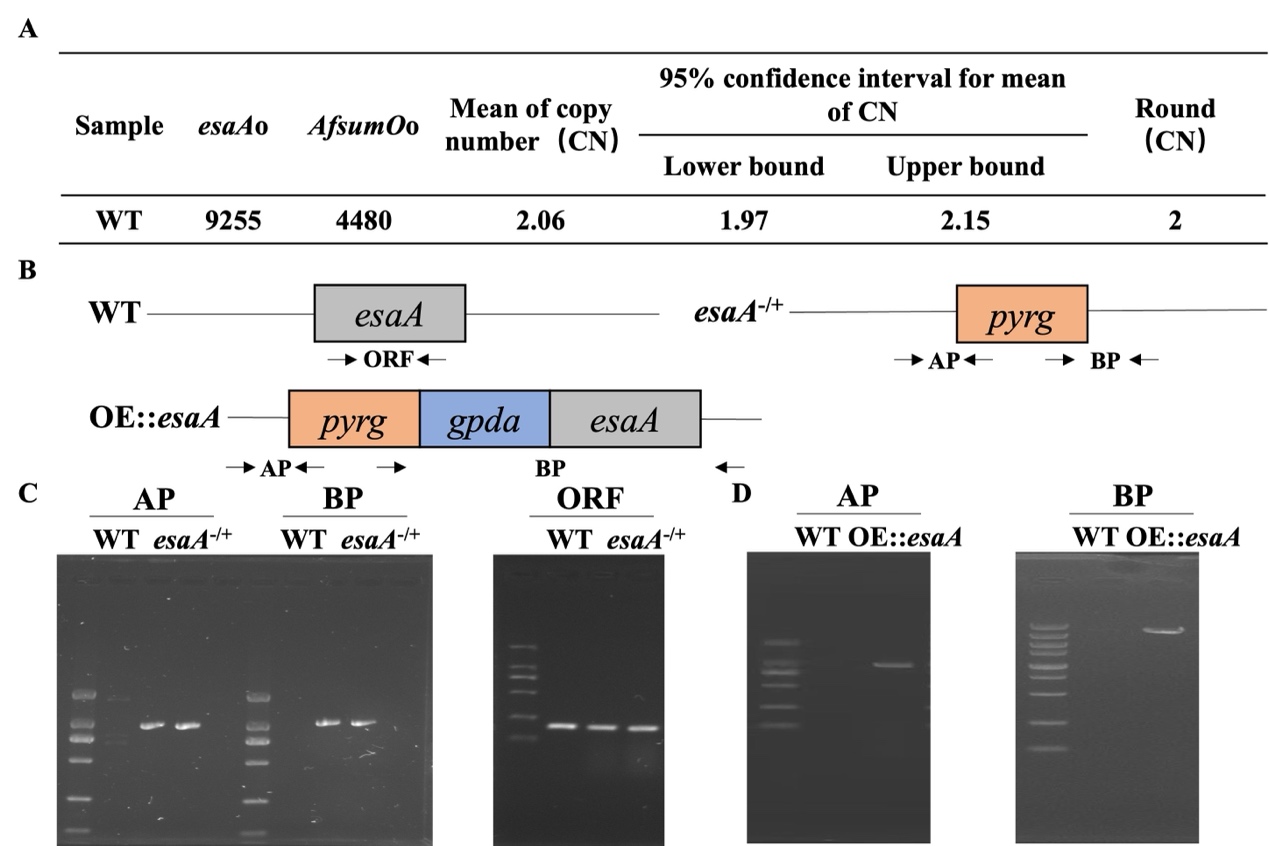


**Fig S12. EsaA catalyzes benzoylation in a picNuA4 complex-independent manner.**

(A) Genomic PCR validation of the upstream homology arm (AP), downstream homology arm (BP) and gene open reading frame (ORF) of the Δ*eaf6*, Δ*yng2* and *epl1*^xylP^ mutants. (B) Western blot analysis of Kbz in WT, Δ*eaf6*, Δ*yng2* and *esaA*^-/+^ strains. (C) Western blot analysis of Kbz in WT, *epl1*^xylP^ and OE::*esaA* strains.


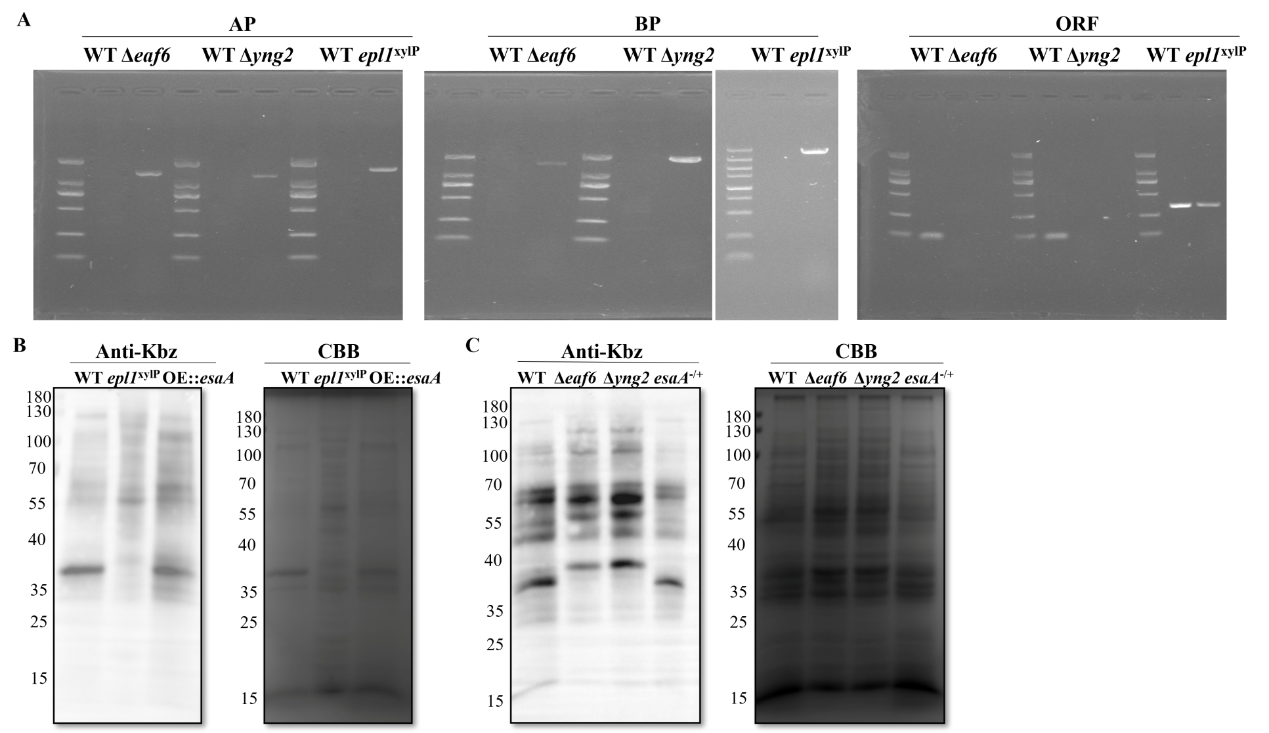


**Fig S13. Purification of EsaA protein.**

Purification of EsaA using Ni-NTA column chromatography. M: protein marker; Line 1: sediment of protein; Line 2: supernatant of protein; Line 3: flow-through fraction from Ni-NTA Column; Line 4-6: elution fractions with Buffer B, C, D, respectively; Line 7-14: elution fractions with Buffer E.


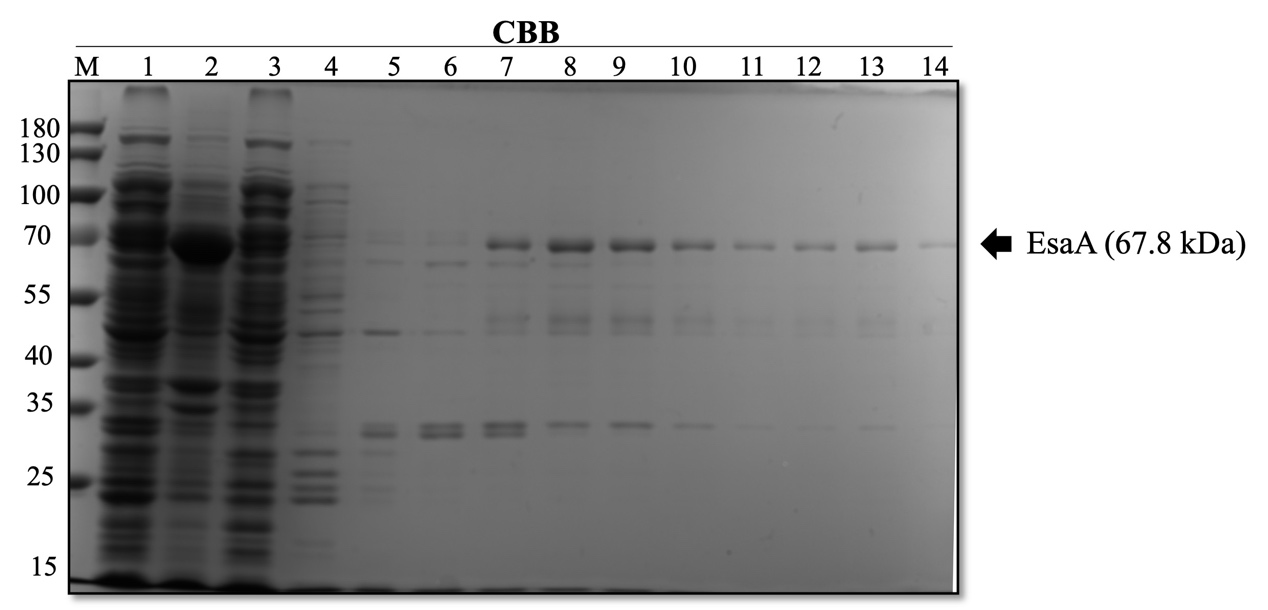


**Fig S14. Construction and confirmation of OE::*esaA*-*foxA*-HA, *esaA^-/+^*-*foxA*-HA and *gcnE*^xylP^-*foxA*-HA mutants.**

(A-C) Genomic PCR verification of OE::*esaA*-*foxA*-HA (A), *esaA^-/+^*-*foxA*-HA (B) and *gcnE*^xylP^-*foxA*-HA (C) mutants. (D-F) WB verification of OE::*esaA*-*foxA*-HA (D), *esaA^-/+^*-*foxA*-HA (E) and *gcnE*^xylP^-*foxA*-HA (F) mutants.

**
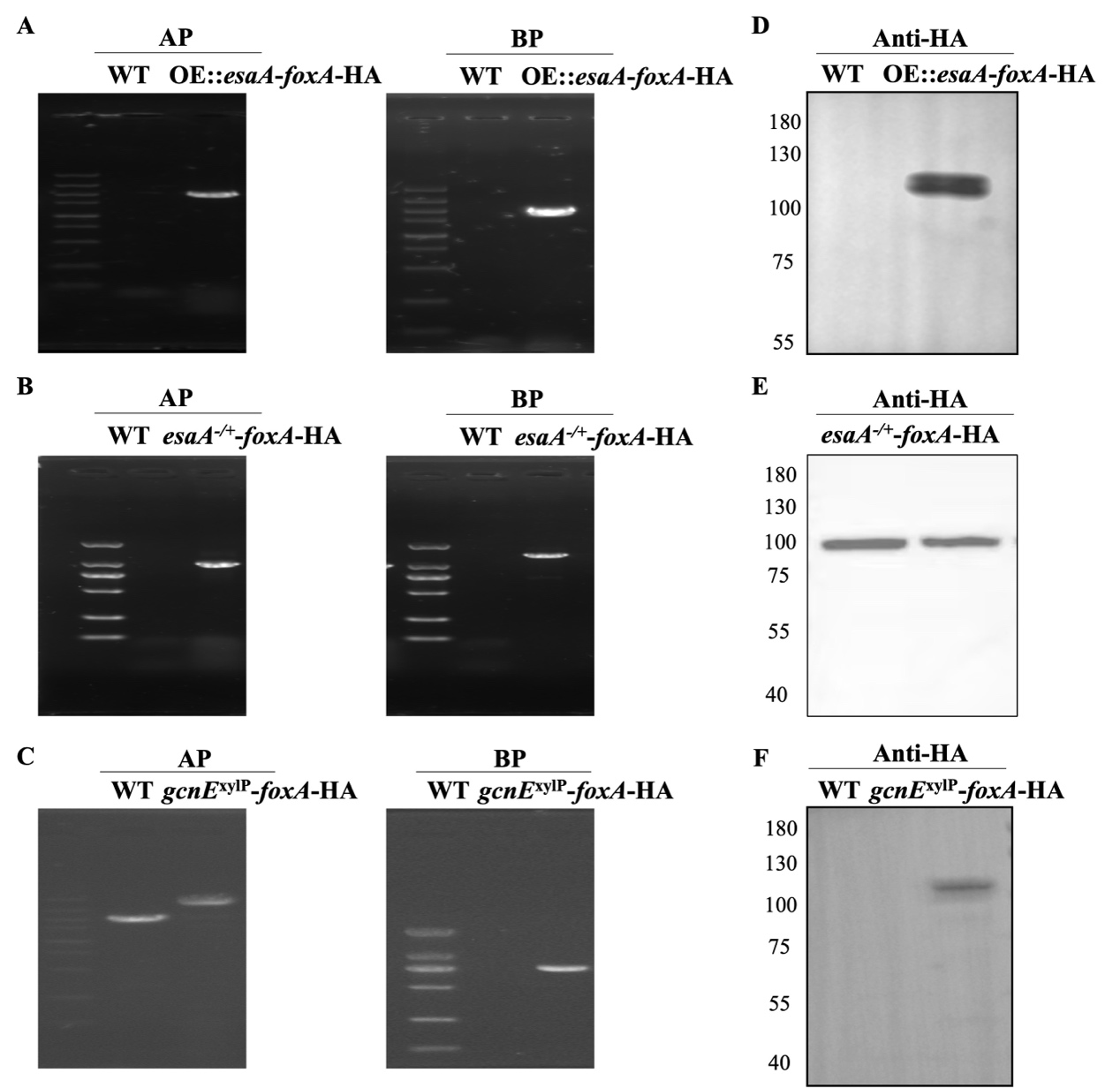
**

**Fig S15. GcnE was not catalyze the benzoylation and acetylation of the FoxA protein.**

Quantitative analysis of FoxA protein Kbz and kac levels in *foxA*-HA and *gcnE*^xylP^-*foxA*-HA strains under repressive (YGT) and inducible (YXT) conditions.

**
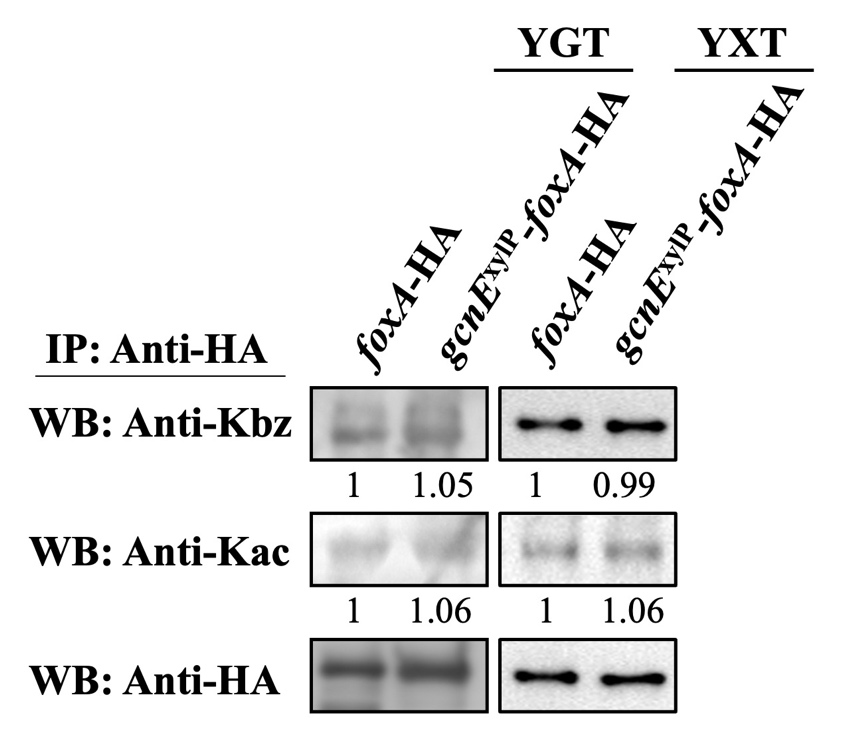
**

**Fig S16. Construction and confirmation of domain and site mutants of EsaA.**

(A) Estimation of copy number of *esaA* gene in *A. flavus*. (B-F) Genomic PCR verification of mutant. (D) Genomic PCR verification of *esaA*^-/+^(B), OE::*esaA* (C), *esaA*^ΔCHO^ (C), *esaA*^ΔMOZ^ (C), *esaA*^E394Q^ (D), OE::*esaA*-*foxA*-HA (E) and *gcnE*^xylP^-*foxA*-HA (F) mutants.

**
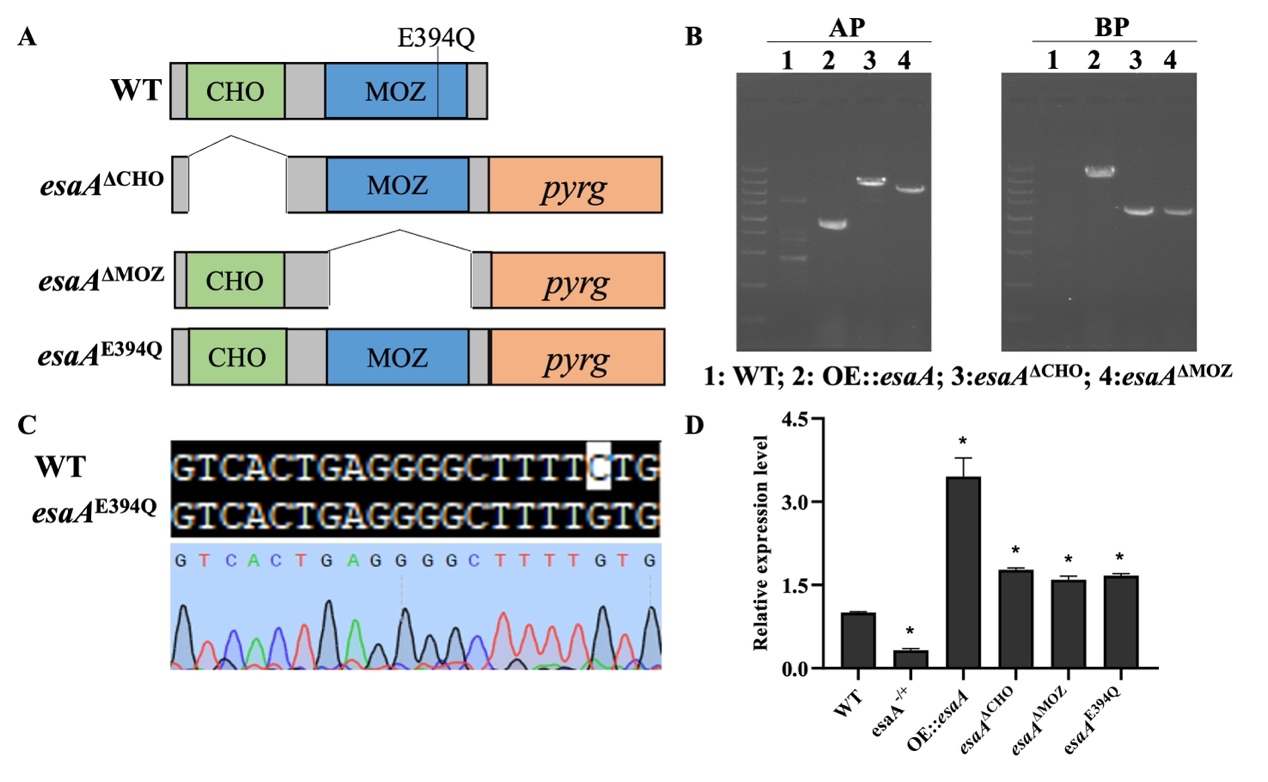
**

**Fig S17. Statistical analysis of EsaA domain mutants.**

(A) Colony diameters analysis of WT and *esaA* mutants (n=3). (B) Amount of conidia produced by the different strains on YGT media (n=3). (C) Relative expression level of *abaA* and *brlA* genes (n=3). (D) Amount of sclerotia produced by different strains on CM media (n=3). (E) Relative expression level of *nsdC* and *nsdD* genes (n=3). (F) Quantification analysis of AFB_1_ in TLC results by optical density (n=3). (G) Relative transcript levels of aflatoxin biosynthesis associated genes in different strains (n=3). Asterisks represent statistically significant differences (P<0.05).


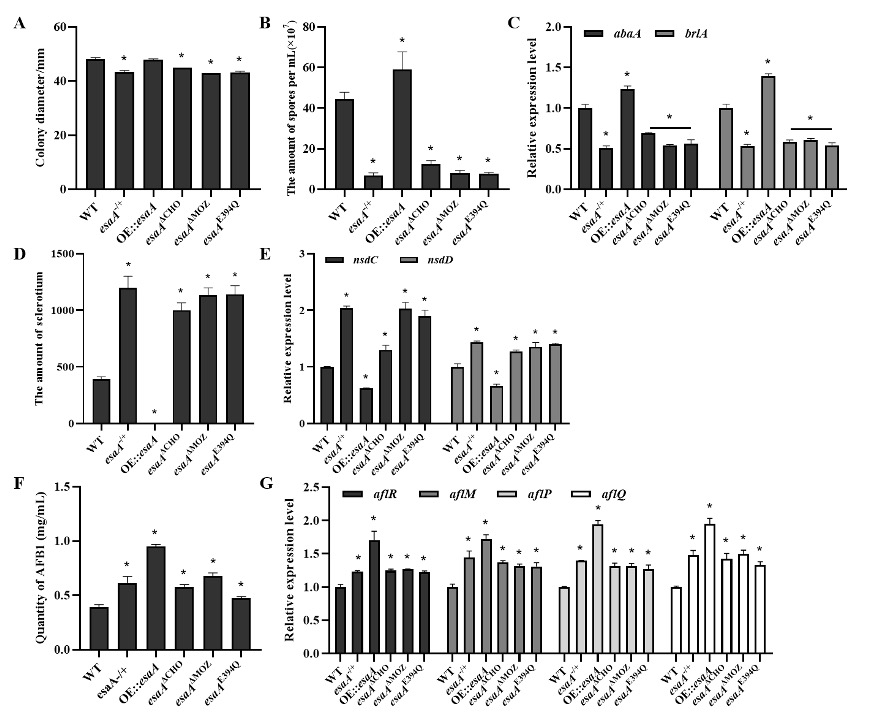


**Fig S18. Quantification of AFB_1_ production in infected maize kernels.**

(A) Phenotypic observation of WT and *esaA* mutants in maize kernels. (B) TLC assay was used to detect the AFB_1_ production extracted from the infected maize kernels (n=3). (C) Quantification analysis of TLC results by optical density (n=3). Asterisks represent statistically significant differences (P<0.05).

**
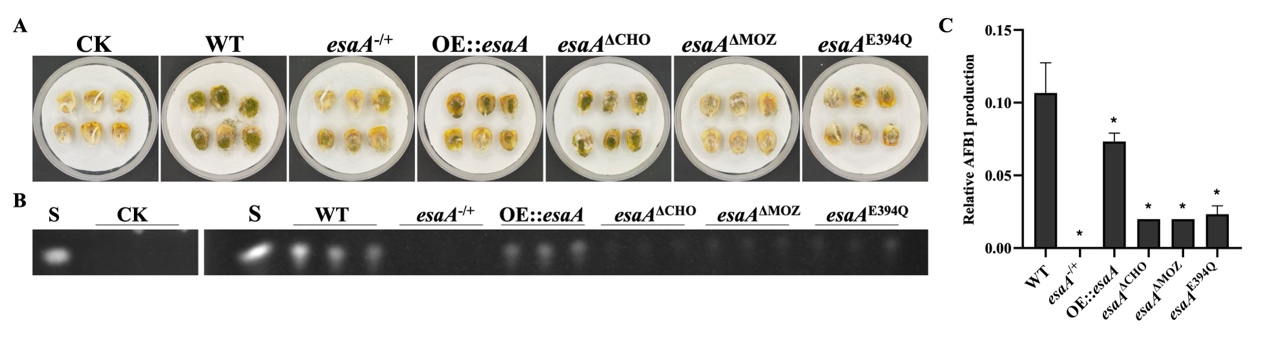
**

**Fig S19. Construction and confirmation of acetylation related *foxA*-HA and *foxA* mutants.**

(A) Sequencing confirmation of acetylation related *foxA*-HA mutants. (B) Sequencing confirmation of acetylation related *foxA* mutants.


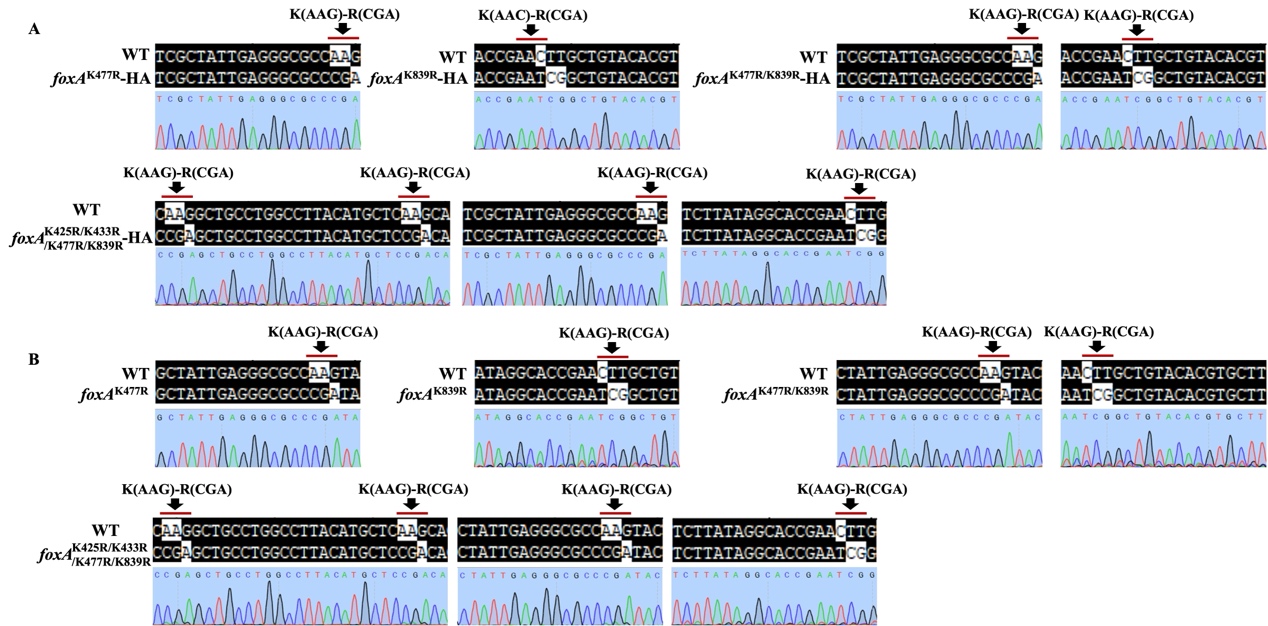


Fig S20. Localization analysis of FoxA protein in acetylated site mutants.


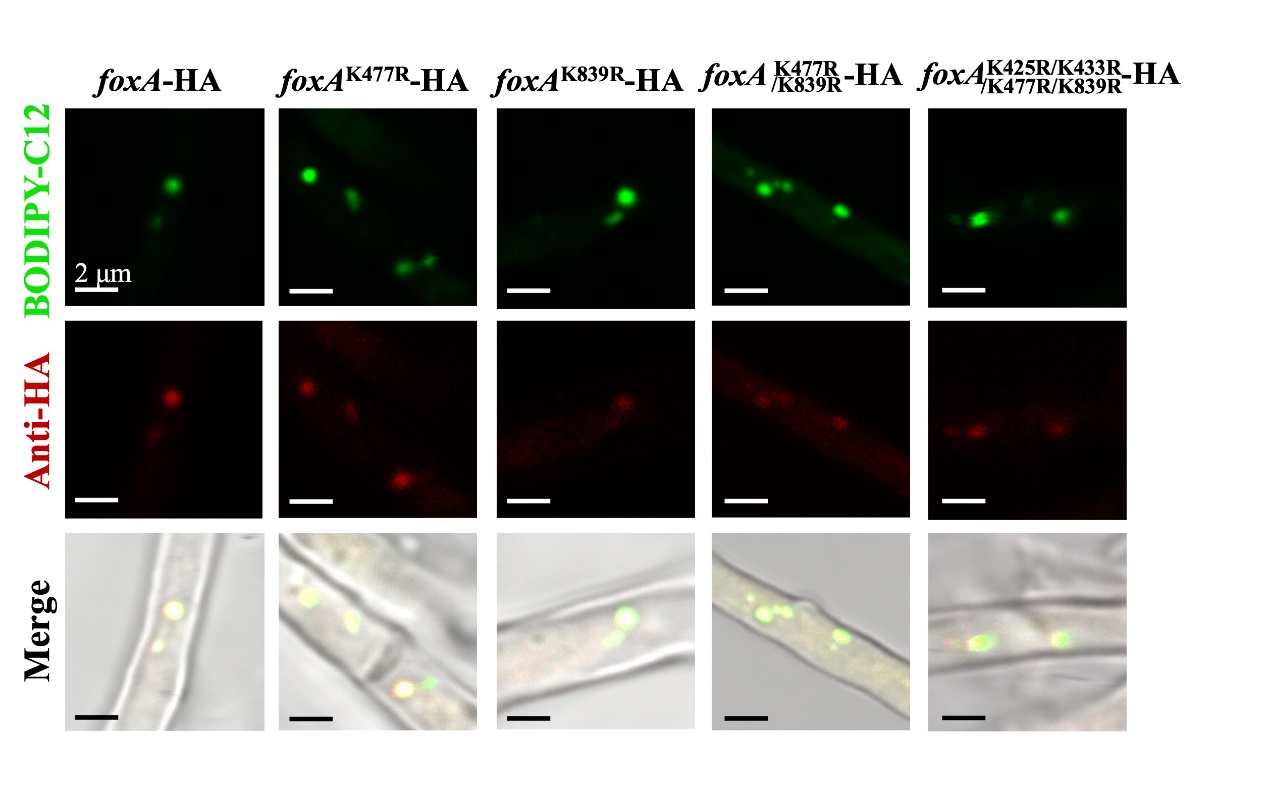


**Fig S21. TLC assay of AFB_1_ production by the WT and FoxA mutants.**

S indicates AFB_1_ standard.


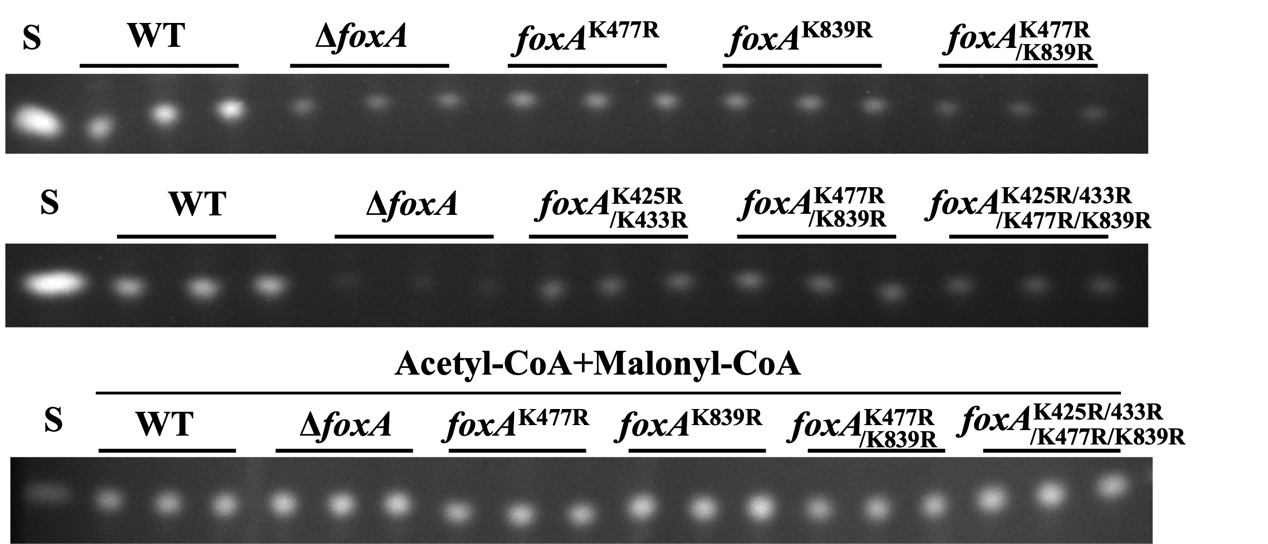


**Fig S22. Kac of FoxA was required for fungal development.**

(A) Colony morphology of WT and *foxA* mutants grown on GMM media and CM media. (B-C) Colony diameter (B), conidia production (C) by different strains on GMM media (n=3). (D) Amount of sclerotia produced by different strains on CM media (n=3). Asterisks represent statistically significant differences (P<0.05).


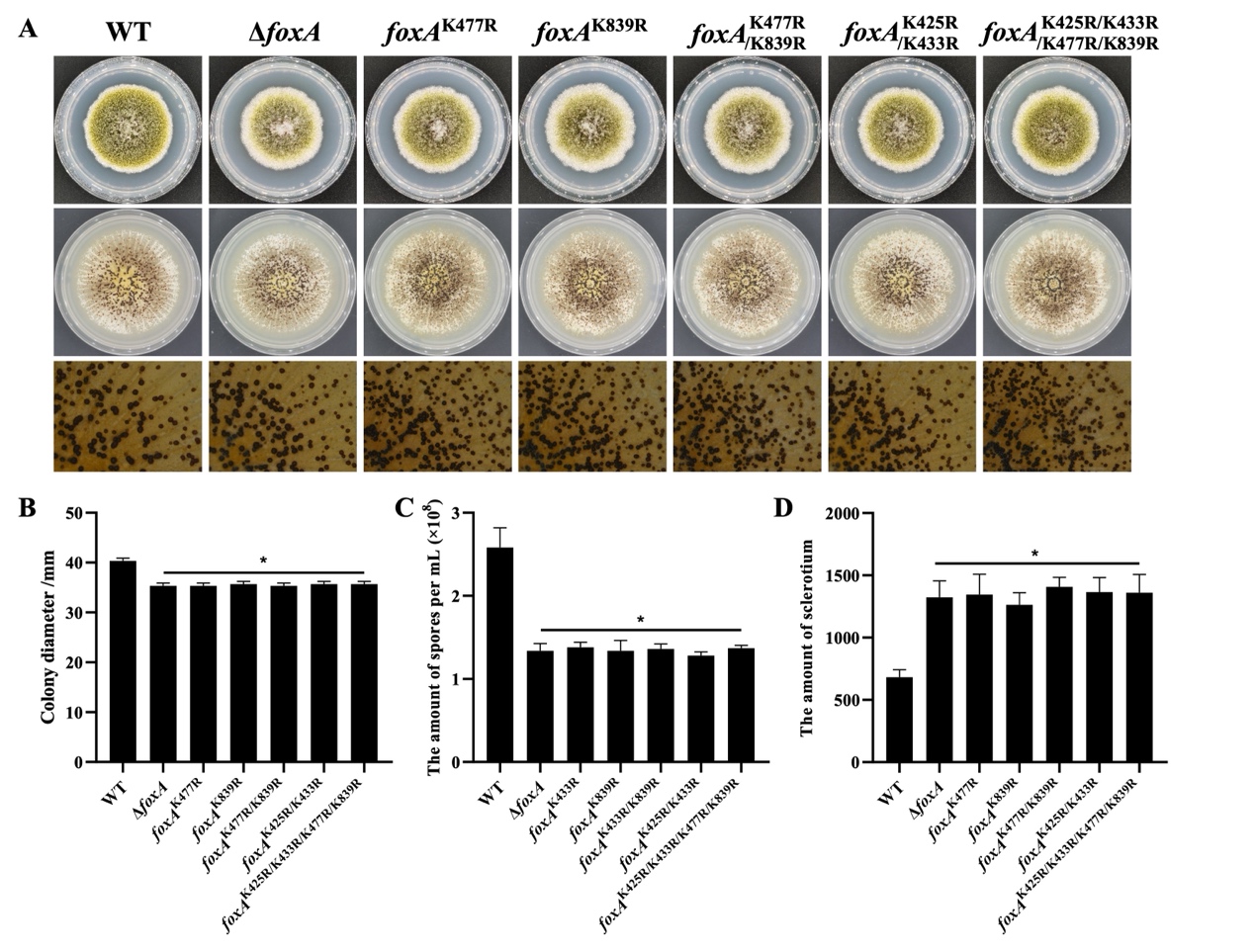


**Fig S23. The Kac of FoxA was important in crop colonization.**

(A) Phenotypic observation of WT and *foxA* mutants in maize kernels. (B) TLC assay was used to detect the AFB_1_ production extracted from the infected maize kernels (n=3). (C) Quantification of conidia from the infected maize kernels (n=3). (D) Quantification analysis of AFB_1_ in TLC results by optical density (n=3). (E) Fungal burdens were estimated by quantification of fungal DNA using qPCR (n=3). Asterisks represent statistically significant differences (P<0.05).


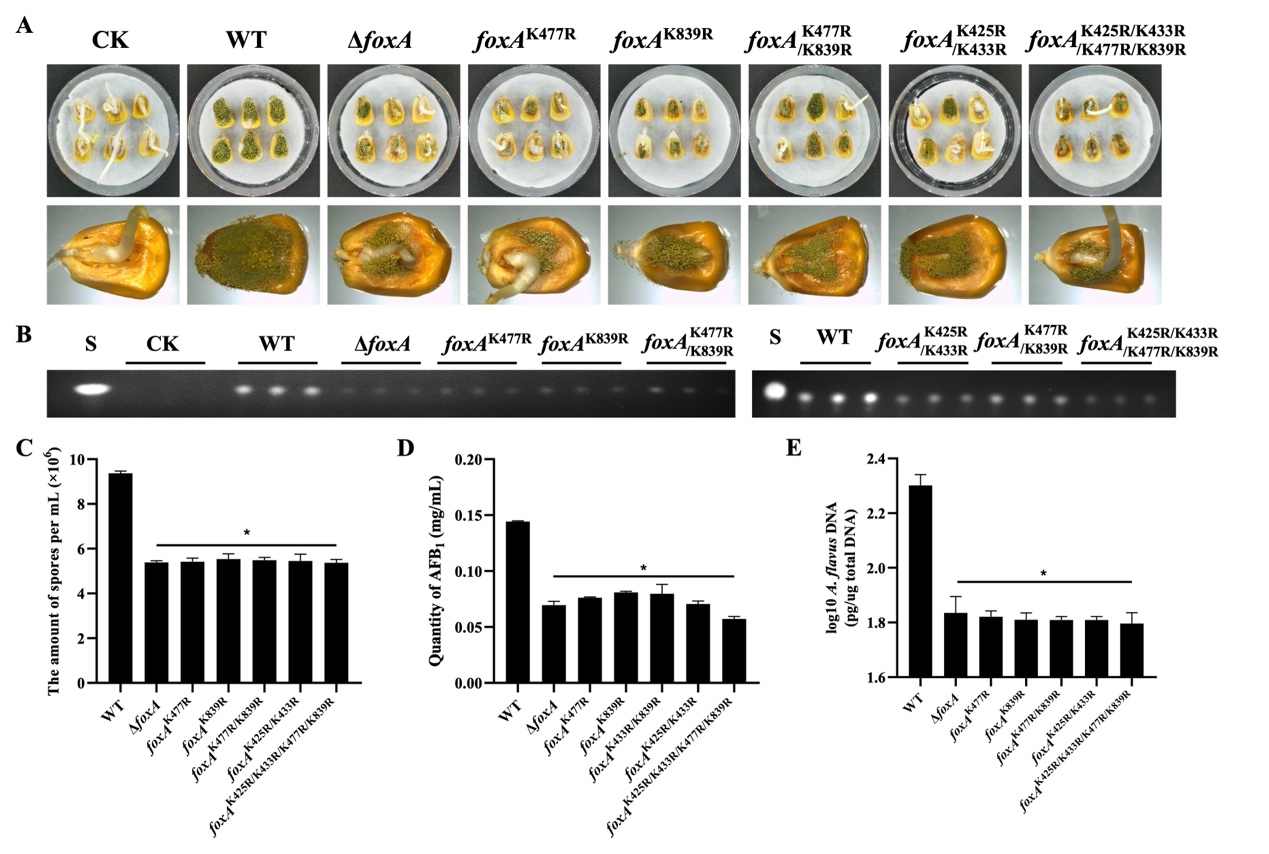


**Fig S24. Predicted structure of the multifunctional protein (MFP) generated by AlphaFold2.**


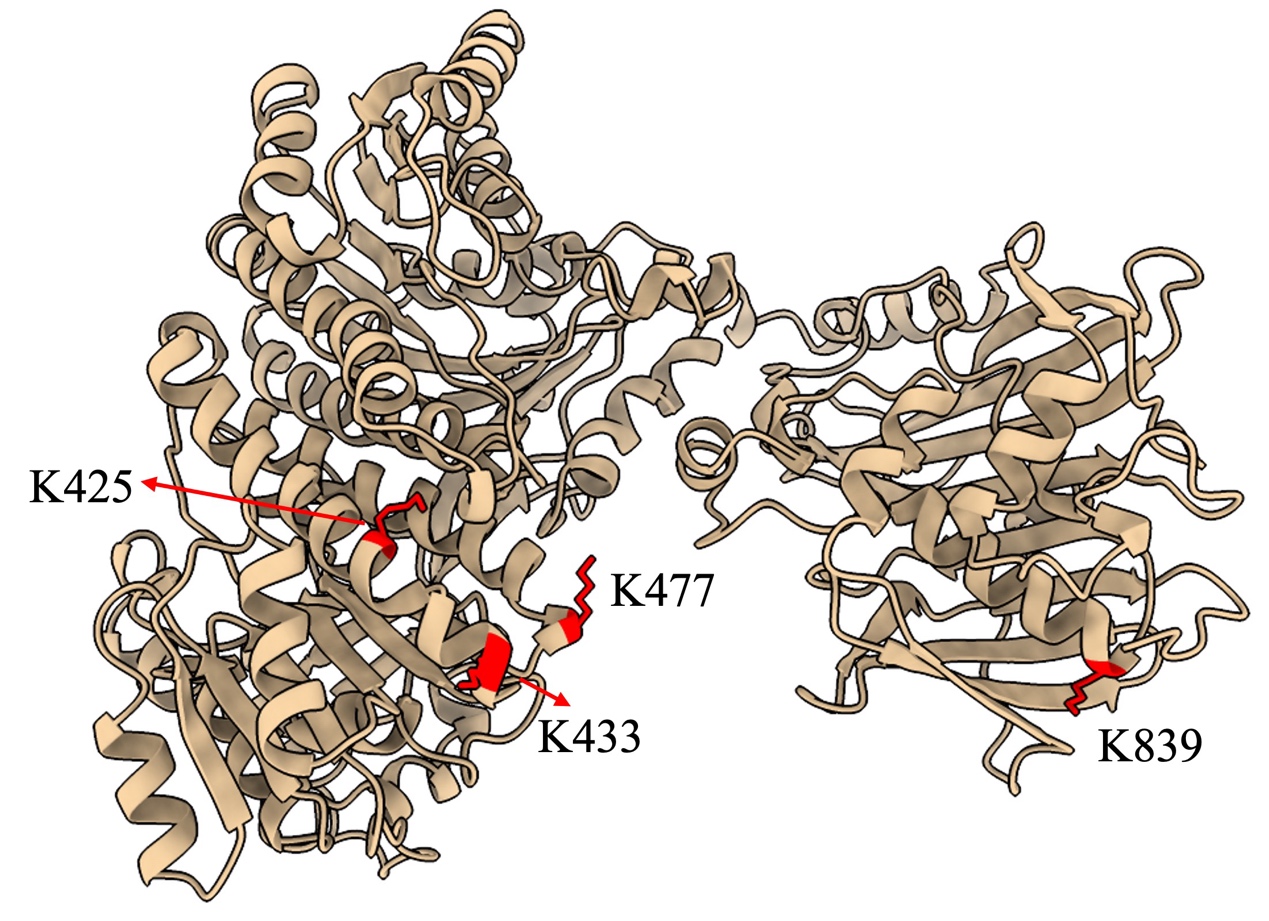


**Table S1. Fungal strains and plasmids are used in this study.**

| Strain and plasmid | Description | Reference |
| --- | --- | --- |
| *A.flavus* CA14PTS | Δ*ku70,* Δ*pyrG* | [60] |
| Wild type | Δ*ku70,* Δ*pyrG*::*AfpyrG* | [58] |
| *foxA*-HA | Δ*ku70,* Δ*pyrG,* Δ*AflfoxA*::*AflfoxA*::*ha*::*pyrG* | This study |
| *foxA*^425R^-HA | Δ*ku70,* Δ*pyrG,* Δ*AflfoxA*::*AflfoxA*^425R^::*ha*::*pyrG* | This study |
| *foxA*^433R^-HA | Δ*ku70,* Δ*pyrG,* Δ*AflfoxA*::*AflfoxA*^433R^::*ha*::*pyrG* | This study |
| *foxA*^477R^-HA | Δ*ku70,* Δ*pyrG,* Δ*AflfoxA*::*AflfoxA*^477R^::*ha*::*pyrG* | This study |
| *foxA*^837R^-HA | Δ*ku70,* Δ*pyrG,* Δ*AflfoxA*::*AflfoxA*^837R^::*ha*::*pyrG* | This study |
| *foxA*^477R/837R^-HA | Δ*ku70,* Δ*pyrG,* Δ*AflfoxA*::*AflfoxA*^477R/837R^::*ha*::*pyrG* | This study |
| *foxA*^425R/433R^-HA | Δ*ku70,* Δ*pyrG,* Δ*AflfoxA*::*AflfoxA*^425R/433R^::*ha*::*pyrG* | This study |
| Δ*foxA* | Δ*ku70,* Δ*pyrG,* Δ*AflfoxA*::*pyrG* | This study |
| *foxA*^425R^ | Δ*ku70,* Δ*pyrG,* Δ*AflfoxA*::*AflfoxA*^K425R^::*pyrG* | This study |
| *foxA*^433R^ | Δ*ku70,* Δ*pyrG,* Δ*AflfoxA*::*AflfoxA*^K433R^::*pyrG* | This study |
| *foxA*^477R/837R/425R/433R^ | Δ*ku70,* Δ*pyrG, ΔAflfoxA::AflfoxA*^K425R/K433R/K477R/K839R^*::pyrG* | This study |
| *foxA*^425R/433R^ | Δ*ku70,* Δ*pyrG, ΔAflfoxA::AflfoxA*^K425R/K433R^*::pyrG* | This study |
| *foxA*-com | Δ*ku70,* Δ*pyrG,* Δ*AflfoxA*::*AflfoxA*::*pyrG* | This study |
| *gcnE*^xylP^*-foxA-HA* | Δ*ku70,* Δ*pyrG,* Δ*AflgcnE::pyrG::xylP::AflgcnE*, Δ*AflfoxA::AflfoxA::ha::pyrG* | This study |
| OE::*esaA*-*foxA*-HA | Δ*ku70,* Δ*pyrG,* Δ*AflesaA*:: *gpdA(p)::AflesaA, ΔAflfoxA::AflfoxA::ha::pyrG* | This study |
| *esaA^-/+^*-*foxA*-HA | Δ*ku70,* Δ*pyrG,* Δ*AflesaA^-^*/*esaA^+^*::*pyrG, ΔAflfoxA::AflfoxA::ha::pyrG* | This study |
| *esaA*^-/+^ | Δ*ku70,* Δ*pyrG,* Δ*AflesaA^-^*/*esaA^+^*::*pyrG* | This study |
| OE::*esaA* | Δ*ku70,* Δ*pyrG,* Δ*AflesaA*:: *gpdA(p)::AflesaA*::*pyrG* | This study |
| *esaA*^ΔCHO^ | Δ*ku70,* Δ*pyrG, AflesaA*^ΔCHO^::*pyrG* | This study |
| *esaA*^ΔMOZ^ | Δ*ku70,* Δ*pyrG, Afl esaA*^ΔMOZ^::*pyrG* | This study |
| *esaA*^E394Q^ | Δ*ku70,* Δ*pyrG, AflesaA*^E394Q^::*pyrG* | This study |
| Δ*yng2* | Δ*ku70,* Δ*pyrG,* Δ*Aflyng2*::*pyrG* | This study |
| Δ*eaf6* | Δ*ku70,* Δ*pyrG,* Δ*Afleaf6*::*pyrG* | This study |
| *epl1*^xylP^ | Δ*ku70,* Δ*pyrG,* Δ*Aflepl1::pyrG::xylP::Aflepl1*::*pyrG* | This study |
| Escherichia coli | DH5α | Takara, Japan |
| Escherichia coli | Rosetta (DE3) | Takara, Japan |
| pET-32a | pET-32a | This study |
| pET-32a-EsaA | pET-32a, Afl*esaA* | This study |

**Table S2. PCR primers used in this study.**

| Primer | Sequence (5’-3’) | Characteristics |
| --- | --- | --- |
| TestF | GCCCCCAATCGTCAAGGTTT | To identification pyrG insertion |
| TestR | AGAGCGCCTTGAGAAAACCAGA |  |
| FoxA-AF | CAAAGACCTCCTTGGCTAATG | To amplify foxA upstream fragment |
| FoxA-AR | GAAGAGCATTGTTTGAGGCCTTGAAACGGGTATAATTAGAAC |  |
| FoxA-BF | GCATCAGTGCCTCCTCTCAGACTGAAACGAGAACTTTTGCGTG | To amplify foxA downstream fragment |
| FoxA-BR | CGAGGGATGATAAACCGAGTC |  |
| FoxA-425KR-AF2 | CAAGGTTACCCGAGCTGC | To amplify K425R mutant site fragment |
| FoxA-425KR-AR1 | GCAGCTCGGGTAACCTTG |  |
| FoxA-425KA-AF2 | CAAGGTTACCGCAGCTGC | To amplify K425A mutant site fragment |
| FoxA-425KA-AR1 | GCAGCTGCGGTAACCTTG |  |
| FoxA-433KR-AF2 | GCTCCGACAGAAATATGGTCG | To amplify K433R mutant site fragment |
| FoxA-433KR-AR1 | CGACCATATTTCTGTCGGAGC |  |
| FoxA-433KA-AF2 | GCTCGCACAGAAATATGGTCG | To amplify K433A mutant site fragment |
| FoxA-433KA-AR1 | CGACCATATTTCTGTGCGAGC |  |
| FoxA-477KR-AF2 | GCCCGATACAACATCAAGGTC | To amplify K477R mutant site fragment |
| FoxA-477KR-AR1 | GACCTTGATGTTGTATCGGGC |  |
| FoxA-477KA-AF2 | GCCGCATACAACATCAAGGTC | To amplify K477A mutant site fragment |
| FoxA-477KA-AR1 | GACCTTGATGTTGTATGCGGC |  |
| FoxA-839KR-AF2 | GTACAGCCGATTCGGTGCCT | To amplify K839R mutant site fragment |
| FoxA-839KR-AR1 | AGGCACCGAATCGGCTGTAC |  |
| FoxA-839KA-AF2 | GTACAGCGCATTCGGTGCCT | To amplify K839A mutant site fragment |
| FoxA-839KA-AR1 | AGGCACCGAATGCGCTGTAC |  |
| FoxA-AR2 | GAAGAGCATTGTTTGAGGCTTACAGTTTAGCCTTGGCTCC | To amplify site mutant upstream fragment |
| FoxA-HA-AR | ACCACTACCTCCGCCACCCAGTTTAGCCTTGGCTCCCTC | To amplify foxA-HA upstream fragment |
| FoxA-NF | TGCTATGACTACGGTGGTTGAG | Nest-primers for fusion PCR |
| FoxA-NR | GAGGTTGAATGAAGTCGGTGG |  |
| gcne-NF | GCCGTTAGTTTGGGCTTAG | Nest-primers for fusion PCR |
| gcne-NR | TTGTGTTCCCGATACCGAC |  |
| gcne-139Q-NF1 | GGAAACAGCAGAAAAGATGGG | Nest-primers for fusion PCR |
| gcne-139Q-NR1 | CTGGCGAATGAGTAAAGTAACG |  |
| gcne-D-AR | GCTTCCGTCGACCTCGAGAGGTTATAACGCTTGCTTGTCAG | To deletion pyrG in gcnE location |
| gcne-D-BF | CTGACAAGCAAGCGTTATAACCTCTCGAGGTCGACGGAAGC |  |
| EsaA-AF | GCGAAGATGCTACGGACTAAG | To amplify esaA upstream fragment |
| EsaA-AR | GGGTGAAGAGCATTGTTTGAGGCCGTGTGGGAGAAGTGATACTG |  |
| EsaA-BF | GCATCAGTGCCTCCTCTCAGACGCGCAAAAATACTCGACCG | To amplify esaA downstream fragment |
| EsaA-BR | CAATGTCCTGTTTCGGGCTC |  |
| EsaA-OE-AR | GGGAATCTCTGACGCCCATCTTAGGGGAAATAAAGGTTCTTGG | To amplify OE::esaA upstream fragment |
| EsaA-OE-BF | CCAAGAACCTTTATTTCCCCTAAGATGGGCGTCAGAGATTCCC | Nest-primers for fusion PCR |
| CHO-A1R | GGACCTGCTTTCTTCTTCTCTCCATCCTAGCGAGGTATGTC | To amplify CHO domain upstream fragment |
| CHO-A2F | GACATACCTCGCTAGGATGGAGAGAAGAAGAAAGCAGGTCC | To amplify CHO domain downstream fragment |
| MOZ-A1R | CCGTGAGGACGATAACCCAGTGAACAAGTGTACATTTCGAACG | To amplify MOZ domain upstream fragment |
| MOZ-A2F | CGTTCGAAATGTACACTTGTTCACTGGGTTATCGTCCTCACGG | To amplify MOZ domain downstream fragment |
| E394Q-AR | CTGAGGGGCTTCTGTGGAGAG | To amplify E394Q mutant site fragment |
| E394Q-BF | CTCTCCACAGAAGCCCCTCAG |  |
| EsaA-A2R | GGGTGAAGAGCATTGTTTGAGGCTCACCAATTCCATGTTCGAC | To amplify E394Q downstream fragment |
| EsaA-D-AR | CAGTATCACTTCTCCCACACGTGCGGAGAGACGGACGGA | To deletion pyrG in esaA location |
| EsaA-D-BF | TCCGTCCGTCTCTCCGCACGTGTGGGAGAAGTGATACTG |  |
| E394Q-NF | CCACGGATTTGGAACCTCAG | Nest-primers for fusion PCR |
| E394Q-NR | CCTCCTTCTACCTTATCAACACTG |  |
| EsaA-NF | GAGGCAGCCATTTCCAGG | Nest-primers for fusion PCR |
| EsaA-NR | TTACCAACCCAGACAAACCAC |  |
| EsaA-32aF | GCCATGGCTGATATCGGATCCCTGGAAGTTCTGTTCCAGGGGCCCATGGGCGTCAGAGATTCC | To amplify esaA-pET-32a fragment |
| EsaA-32aR | GGTGGTGGTGGTGGTGCTCGAGTCACCAATTCCATGTTCGACTTG |  |
| eaf6-AF | CGAGGATAGGTCACCAGAAGG | To amplify eaf6 upstream fragment |
| eaf6-AR | GGGTGAAGAGCATTGTTTGAGGCGTTCGTTGCCTCGCTTTG |  |
| eaf6-BF | GCATCAGTGCCTCCTCTCAGACACTCGATTTTGAGGGAGTTTTG | To amplify eaf6 downstream fragment |
| eaf6-BR | CACTCCAGTAGAACCAGCATCG |  |
| eaf6-NF | GGTCTTCCCCTCGAAATAGTTG | Nest-primers for fusion PCR |
| eaf6-NR | GAAAATCGCCGTCCACTATG |  |
| yng2-AF | GTACTTCAGGACGGCACATTG | To amplify yng2 upstream fragment |
| yng2-AR | GGGTGAAGAGCATTGTTTGAGGCCGATGTTGTTCTCAAGCGACC |  |
| yng2-BF | GCATCAGTGCCTCCTCTCAGACTCATACCCTCCAGTTTATCTTGC | To amplify yng2 downstream fragment |
| yng2-BR | GACTCGGTTTCATCTCGTTG |  |
| yng2-NF | ACTGCGGATAAACCTCCTGTC | Nest-primers for fusion PCR |
| yng2-NR | GAAAAGTATGCCATTGTTCTCTAGG |  |
| EPL1-P-AF | AACTGACATCCGTATTCTCCACC | To amplify epl1 upstream fragment |
| EPL1-P-AR | GGGTGAAGAGCATTGTTTGAGGCGCGAACTTTGGGAGCACGA |  |
| EPL1-P-BF | CATTCATCGACTCGAAGAACCAACATGACTAGGTACGGGGGAATG | To amplify epl1 downstream fragment |
| EPL1-P-BR | TTTTGGTTGCTGACCTCATAG |  |

**Table S3. qPCR primers used in this study.**

| Primer | Sequence (5’-3’) | Characteristics |
| --- | --- | --- |
| brlA-QF | GCCTCCAGCGTCAACCTTC | To detect the *brlA* gene transcript level |
| brlA-QR | TCTCTTCAAATGCTCTTGCCTC |  |
| abaA-QF | CACGGAAATCGCCAAAGAC | To detect the *abaA* gene transcript level |
| abaA-QR | TGCCGGAATTGCCAAAG |  |
| wetA-QF | GCGTCTAGTTGTCAGGAG | To detect the *wetA* gene transcript level |
| wetA-QR | GCGGAGACAGAGATGGTG |  |
| nsdC-QF | GCCAGACTTGCCAATCAC | To detect the *nsdC* gene transcript level |
| nsdC-QR | CATCCACCTTGCCCTTTA |  |
| nsdD-QF | CGGAACCAGGATGCCTTACTAC | To detect the *nsdD* gene transcript level |
| nsdD-QR | CTGCTTTGCCTCGTCGCTTC |  |
| aflR-QF | AAAGCACCCTGTCTTCCCTAAC | To detect the *aflR* gene transcript level |
| aflR-QR | GAAGAGGTGGGTCAGTGTTTGTAG |  |
| aflM-QF | ATCCTGACCAGCTCTAACAC | To detect the *aflM* gene transcript level |
| aflM-QR | ATCTTCTTGTCTCCGCAGTC |  |
| aflN-QF | TTCATTCCTGAGCGATGG | To detect the *aflN* gene transcript level |
| aflN-QR | CGTATGCTGGCGTAATATC |  |
| aflP-QF | ACGAAGCCACTGGTAGAGGAGATG | To detect the *aflP* gene transcript level |
| aflP-QR | GTGAATGACGGCAGGCAGGT |  |
| aflQ-QF | GTCGCATATGCCCCGGTCGG | To detect the *aflQ* gene transcript level |
| aflQ-QR | GGCAACCAGTCGGGTTCCGG |  |
| foxA-QF | TGACTGCCACCATTATGCCTC | To detect the *foxA* gene transcript level |
| foxA-QR | ACCGCCGACTTCGTAGATTC |  |
| esaA-QF | CGTACAAGCGGAAGTATGACC | To detect the *esaA* gene transcript level |
| esaA-QR | GAACGAGGCAGGATAAGGAG |  |
| eaf6-QF | GATTCGACGCCATCATCTGTTC | To detect the *eaf6* gene transcript level |
| eaf6-QR | ATCACCATTCGGTTTCCCTG |  |
| yng2-QF | ATAGCGACCAATACCGAGACC | To detect the *yng2* gene transcript level |
| yng2-QR | ACGTAGGATGGAGGGGAGAG |  |
| epl1-QF | GAAGAAGAGGTCCAGAATGCC | To detect the *epl1* gene transcript level |
| epl1-QR | GCAGGTTGCGAGAAGATAGG |  |
| lys20-QF | GTCGTCATCGGCACTTCGTC | To detect the *lys20* gene transcript level |
| lys20-QR | GCAATACCAACACGGTTCACAC |  |
| asdA-QF2 | GCTGTGATTCCTCAGGCTACG | To detect the *asdA* gene transcript level |
| asdA-QR2 | GAGAAGCAATCCATCCCACG |  |
| shm1-QF | GTGCCAGATACTACGGAGGAAATG | To detect the *shm1* gene transcript level |
| shm1-QR | GTGTTGAGAAGGGCGGAGATG |  |
| trp3-QF | TTTGATGGGCTACTACAACCCTATG | To detect the *trp3* gene transcript level |
| trp3-QR | CTGAAACGCACAGCTTCCTCG |  |
| hisF-QF | GGGAATGTTCGTTCGTTGG | To detect the *hisF* gene transcript level |
| hisF-QR | GAATCTATGTGCTTCTTTATCGG |  |
| aldo-QF | GGGTTGACTATCTGGCTCCG | To detect the *aldo* gene transcript level |
| aldo-QR | AGACACCTGCTTCGATACACGC |  |
| tyrB-QF | GAAAGTTGTTCCCGCTATTTGATG | To detect the *tyrB* gene transcript level |
| tyrB-QR | CTGCGAAGAAAAGACAGCCCAC |  |
| thrB-QF | GTAAATCCTATTCCTCTGGGTCG | To detect the *thrB* gene transcript level |
| thrB-QR | TGAGTTCATTCAAGTAAGTTCCG |  |
| actin-QF | ACGGTGTCGTCACAAACTGG | To detect the *actin* gene transcript level |
| actin-QR | CGGTTGGACTTAGGGTTGATAG |  |
